# Natural language processing captures memory content associated with shared neural patterns at encoding and retrieval

**DOI:** 10.1038/s44271-026-00481-0

**Published:** 2026-06-04

**Authors:** June-Kyo Kim, Joshua Koh, Charan Ranganath, Alexander J. Barnett

**Affiliations:** 1 University of Toronto, Department of Psychology, Toronto, Canada; 2 McGill University, Department of Neurology & Neurosurgery, Montreal, Canada; 3 University of California, Davis, Center for Neuroscience, Davis, USA; 4 McGill University, McConnell Brain Imaging Centre, Montreal, Canada

**Keywords:** Human behaviour, Cognitive neuroscience

## Abstract

People can experience the same event yet form distinct memories shaped by individual interpretations. Prior research shows that multivariate activity patterns in the Default Mode Network (DMN) are correlated across individuals during shared experiences, suggesting a role in representing high-level event features. However, it remains unclear whether these shared neural patterns reflect similarity in subsequent memory content. Here, we examined whether memory similarity correlates with intersubject spatial patterns in the DMN using a pre-existing dataset. Twenty-four individuals watched and recounted two cartoon movies during fMRI scanning. Using topic modeling, we transformed verbal recall into vectors of latent topics to quantify memory similarity across participants. We found that greater similarity in recalled content was associated with stronger shared activation patterns at encoding and retrieval, particularly in the posterior medial, medial prefrontal and anterior temporal cortices. These findings highlight the utility of natural language processing tools in linking memory representations to brain activity and underscore the DMN’s role in encoding, interpreting, and recalling complex event features.

## Abstract (web summary)

This study shows that greater similarity in recalled content was associated with stronger shared activation patterns at encoding and retrieval in prefrontal and temporal cortices.

## Introduction

Narratives unify shared experiences between people and are thought to be adaptive for group cooperation^1^. However, even when watching or reading the same narrative passage, people may notice unique details or find special significance in some elements of the story compared to others. These shared and unique elements shape how we remember our experiences.

Previous studies have found that encoding and retrieval of narratives drives shared multivariate patterns in default mode network (DMN) regions among people watching or recalling the same stories^2,3^. These patterns are event specific suggesting that memory representations for real-world events are spatially organized in a manner that is generalizable across brains^4^.

Neocortical DMN regions are thought to represent higher-order, abstracted event models of ongoing situations^5–7^. Further, this collection of regions has been shown to functionally couple with each other and the hippocampus at rest to form a large-scale intrinsic network^8,9^ and interactions of these regions with the hippocampus during encoding and retrieval are thought to reflect the storage and reactivation of event-specific information^10–13^.

Prior research has shown that individuals who share the same interpretation of a narrative had greater inter-subject synchrony of their timecourses in the majority of the DMN^14,15^, and have greater correlations in their multivariate patterns^16^ compared to those with an opposing interpretation. This work has linked the medial prefrontal cortex (mPFC), posterior medial cortex (PMC) and anterior temporal lobes (ATL) with higher order event representation as multivariate neural patterns during recall were more correlated when participants recalled events with the same interpretation^16^. These findings are in line with theories that the DMN represents abstract event models^7,11^.

Building from these findings, one might expect that people who have similar DMN event representations at encoding may go on to recall similar information at retrieval. To our knowledge, no study to date has examined this relationship. Further, while previous studies have shown that people with similar interpretations at recall have similar DMN events patterns, these studies compared participants in discrete groups by experimentally manipulating their interpretations of events^15,16^. These manipulations involved either selectively providing or withholding information to bias the meaning of events (e.g. one group gets to see the end of a narrative which reframes how earlier events are viewed). However, such approaches risk oversimplifying the multifaceted nature of narrative memory. For example, one pair of subjects may share many aspects of their recall, another pair may share a few, and another pair may have opposing views of an event. Recent advances in natural language processing models have provided powerful tools for analyzing the language people use during recall^14^. These models can uncover latent themes in spoken or written recollections, allowing researchers to identify both shared and unique aspects of remembered content^17^. By examining which combinations of latent themes are common across pairs of participants, this approach enables a more nuanced assessment of the similarity in their memory representations.

In this study, we used natural language processing and fMRI to examine how multivariate event patterns in the DMN are shared during encoding and recall, depending on the degree of similarity in which participants remembered events. To do so, we re-analyzed data from Barnett et al.^10^ in which 24 participants watched and verbally recalled two cartoon movies during fMRI scanning. We hypothesized that people who recall events more similarly to each other would show stronger intersubject pattern similarity in DMN regions, particularly the mPFC and PMC – regions that have been heavily implicated in representing higher order abstractions of events^7,16,18,19^. We also examined how recall similarity related to simpler scoring measures of memory content such as the total number of details recalled and the number of intrusions, expecting greater similarity in recall when participants recalled many true details and few intrusions.

## Methods

### Dataset

Our dataset is a re-analysis of Barnett et al.^10^ which consisted of 24 healthy, right-handed young adults (mean age = 23.5 years; N_Female_ = 14, N_Male_ = 10) with no history of neurological issues from the local Davis, California community. Participants self-reported sex. Communities of descent, race, and socioeconomic status were not recorded. After giving informed consent, participants watched two 16.5 min custom-made cartoon movies and then freely recalled them in the scanner^10^. These custom-made cartoons were made using Plotagon Studio (https://www.plotagon.com/desktop/). The movies depicted a continuous narrative, one following a stereotypical crime scene investigation style story and the other depicting a medieval fantasy style story. Each movie consisted of 14 events (mean 72 s in length) which were defined by shifts in location, time, and character composition. These events were confirmed and identified by a sample of 62 online participants from Barnett et al.^10^ who were instructed to watch one of the videos and press the spacebar when they felt that “one meaningful event had ended and another had begun” in line with prior work in the field^20,21^. The resulting timecourse of button presses from each participant was combined to form a distribution which was modelled with a kernel density estimate. Peaks in the density confirmed the intended event boundaries designed in the stimuli (and identified an additional boundary in each stimulus). These were used as “event boundaries” in Barnett et al.^10^. Here, an “event” is defined as the time between one event boundary and the next. Participants were compensated $20 an hour for their time and the study was approved by the institutional review board of the University of California at Davis (IRB #1352490 & IRB # 637028) and the University of Toronto (45451). This study was not preregistered.

### MRI acquisition

MRI data was collected at both encoding and recall using a 3T Siemens Skyra scanner system with a 32-channel head coil. Two T1-weighted structural images were acquired using a magnetization prepared rapid acquisition gradient-echo (MPRAGE) pulse sequence (TR = 1900 ms) and functional images were acquired using a gradient EPI sequence (TR = 1220 ms; TE = 24 ms; field of view = 192 mm^2^; image matrix = 64 × 64; flip angle = 67°; multiband factor = 2; 38 interleaved axial slices, voxel size = 3 mm^3^ isotropic). For detailed scanning parameters see Barnett et al.^10^. Participants viewed and verbally recalled each video in turn during fMRI. During viewing, participants were instructed to watch the movies as if they were a television show they were interested in. Without cues, participants freely recalled the movies aloud using an MRI compatible microphone. They were instructed to “In as much detail as possible tell me everything you can remember about the last movie we showed you. Try to recount the events in the original order in which they occurred. Completeness and detail are more important than temporal order. If at any point you realize that you have missed something, return to it. Try to describe EVERY detail that you have about the movie you just watched, even if it seems irrelevant”. Participants verbally recalled into an MRI compatible microphone (Optoacoustics FOMRI-III; https://www.optoacoustics.com/medical/fomri-iii) with bidirectional noise-cancelling to mitigate scanner noise. Participants were allowed to recall until they could no longer recall any additional details. Thus, run lengths varied between participants from 108 s to 1016 s (mean = 688 s).

### Recall scoring

Audio recall for each participant was transcribed and scored. First, each participants transcript was divided into events based on the content being described by trained raters. For example, if the participant said “In the next scene, Arlene and Muller talked outside in the burning village …” then this and the text that immediately follows would be classified as event 13—the penultimate event of one of the movies. This event would be classified as finished when the participant changed to describing a different event (e.g. “Next we cut to the ballroom where we see the Count waiting for Muller’s return”). Two trained raters then scored each event for the number of details recalled. Details were defined as “a unique occurrence, observation, or thought, typically expressed as a grammatical clause. Additional information in the clause was scored separately” as defined by Levine et al.^22^. For example, “they wanted to talk privately…” is one detail and “…so they went inside the bar” is another detail. Only details that were verifiably true according to the events depicted in the movies were considered. We then calculated the sum of these verifiable details recalled for each event. In some cases, participants would recall a detail from another event during the recounting of an event. These details were classified as intrusions. For additional information on scoring and labeling procedures, see ref Barnett et al.^10^.

### Preprocessing

Initial preprocessing was done using fMRIPrep^23^. See Barnett et al.^10^ for full details. Anatomical preprocessing included skull stripping, brain tissue segmentation, cortical surface reconstruction using FreeSurfer^24^, and volume-based spatial normalization to standard space. Functional images were corrected for susceptibility distortion and were co-registered to the T1w reference image and then resampled into standard space. Regressors of no interest including six-degree motion parameters and motion outliers (framewise displacement >0.5 mm, DVARS > 3) were regressed out of the data and the data was high-pass filtered to remove frequencies below 0.001 Hz all in one step using tproject^25^ in nipype^26^.

### Intersubject pattern similarity analysis

**Fig. 1:**
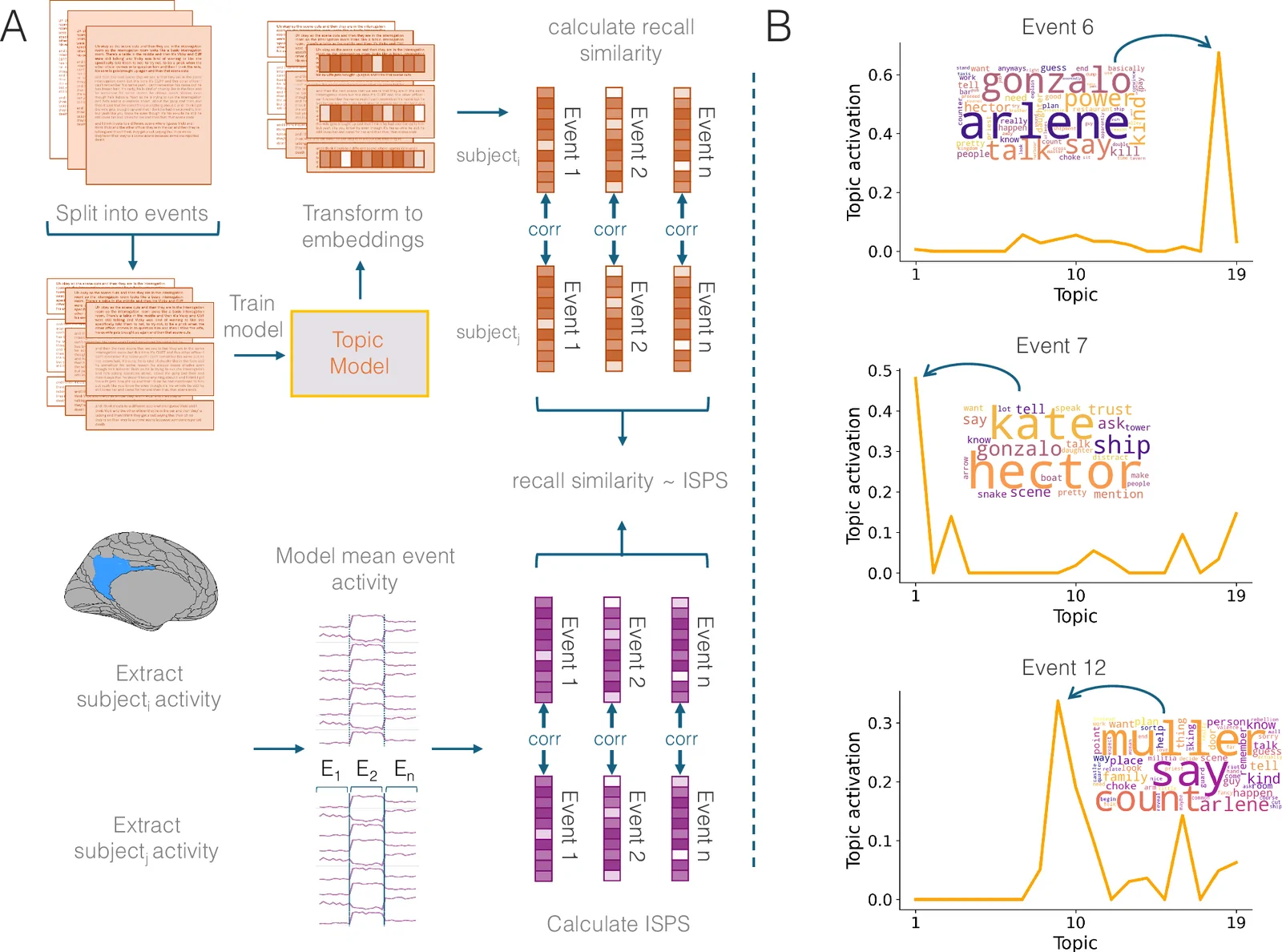
Overview of analysis pipeline. **A** TOP: The preprocessed recall transcripts for each participant were divided into separate documents for each event. The collection of documents was then used to train a topic model to identify latent topics. Each event from each participant was then transformed into a vector of topic proportions that reveal the combination of topics present in each subject’s recalled events using the trained topic model. To calculate topic similarity, we correlated the vector of topic proportions from matching events between participants and Fisher z-transformed the correlation. BOTTOM: To examine intersubject pattern similarity (ISPS), multivoxel activity was extracted from each ROI and was modeled to find the mean multivoxel pattern for each event and each participant. We then correlated these multivoxel patterns for matching events between pairs of participants and Fisher z-transformed the correlation to get ISPS. **B** Plots showing the average topic embeddings for three example events along with word clouds highlighting the top words that contribute to those latent topics (larger words are weighted more highly in a topic). Of note, topics often highly weighted the presence of character names, spatial locations, or topics of conversation (e.g., Hector, Kate, Ship, for event 7 that depicts those characters Hector and Kate on a boat).

To examine multivoxel patterns, we present both a predefined ROI approach in which we assess the contributions of regions of the DMN alongside an exploratory whole-brain approach. For the whole brain, we used 360 parcels from the HCP-MMP1.0 atlas^27^. For the pre-defined ROI approach, we constructed 10 ROIs by combining spatially contiguous regions of the DMN that were previously found to cluster into DMN subnetworks using intrinsic functional connectivity^8^ from the HCP-MMP1.0 atlas^27,28^. The 10 ROIs include the PMC, medial temporal lobe (MTL), precuneus (PreC), ventral mPFC (vmPFC), dorsal mPFC (dmPFC), ATL, ventral lateral parietal (vLP), dorsal lateral parietal (dLP), anterior lateral parietal (aLP), and a control region, the somatomotor cortex (SMC) that we did not predict would track abstract internal event representations. The combination of regions for each ROI can be found in Supplemental Table 1. The corrected voxel timecourses of each region (or combination of regions) were extracted then z-scored across the entire run (Fig. 1). Mean multivoxel event activity was estimated by modeling the data with time blocks for each event convolved with AFNI’s hemodynamic response function^25^. A multivoxel activity pattern was created per event, per subject, and per region using linear regression to estimate the mean event activity. For every pair of participants, we calculated the Pearson’s correlation between event activity patterns of each matching event for every region of interest. These correlation values were Fisher z-transformed and used as the dependent variable, intersubject pattern similarity, in linear mixed models described below.

### Natural language processing

To quantify the similarity of people’s memory recall, the participant recall transcripts were used to perform topic modeling. We performed initial preprocessing including lemmatization and removing stop words using scikit-learn 1.4.1^29^, manually removed punctuation, and standardized capitalization as done in Heusser et al.^17^. Additionally, we normalized the spelling of names or places in transcripts. For example, one participant’s recall may have been transcribed to call the main character Arlene and another participant’s recall may have been transcribed as Ailene. In this situation, we would convert all mistranslated names to the standard character’s name, Arlene. For each participant, we divided the recall transcript into separate segments based on the event that was being described. Independent raters read through each transcript and delineated the moments when a participant was referencing a specific event as defined in our previous paper^10^. Each delineated collection of recall for events served as “documents” in topic modeling.

Using methods developed by Heusser et al.^17^, we trained our model using these recall documents with scikit-learn 1.4.1^29^, called from HyperTools^30^. Broadly, we used the CountVectorizer class, to transform the words from the collection of documents into a vector of word counts yielding a document-by-word count matrix. For the collection of documents, we submitted the preprocessed recall transcripts for each event and each participant (Fig. 1). We used LatentDirichletAllocation (LDA) class to train the topic model to the word count matrices, which identified latent themes within the documents. We could then apply the model to the data of each participant to evaluate the presence of each latent theme within each event, resulting in an event-by-topic proportion matrix per participant. Each event vector in these matrices describes the mixture of discovered topics (latent themes) present within the event. Using the event-by-topic proportion matrix as a proxy for recalled content, recall similarity for each event was estimated by correlating event-specific topic vectors between every pair of participants using Spearman correlations. All correlations were Fisher z-transformed to make a measurement we call *topic similarity*.

When training a topic model, certain parameters can affect the model, like the number of topics (*K*). Given that we didn’t have a strong prior regarding the number of topics, we performed a parameter sweep from 5 to 59 topics spaced by 2. To optimize the number of topics, at each topic *K*, we split the data into a training dataset (80% of the data) and a testing data set (20%). A topic model was fit to the training dataset and used to transform both the training and testing dataset into their constituent event topic vectors. A pattern classifier was then trained on the training dataset to predict event labels based on event topic vectors and tested on the left-out testing dataset using multinomial logistic regression with L2 penalty. This was done for each movie separately and repeated 30 times at each topic *K*. Model accuracy was plotted against topic number to identify inflection points in model performance where increasing *K* resulted in diminishing returns. Next, we examined the latent topics identified from the LDA to evaluate their interpretability and their redundancy. After selecting the optimal *K*, we calculated topic similarity. The fitting of the topic model will vary based on a random seed, resulting in slightly different models each time. To ensure that our estimates of topic similarity are not sporadic, we also fit the model 100 times using a new random seed for the optimal *K* number of topics and averaged the similarity to bootstrap our findings and used the average topic similarity. Intraclass correlation was calculated across the 100 iterations to determine the impact of the random seeds. The ‘batch’ learning method was used, and all other parameters were kept as default.

### Statistical analyses

First, we examined how the topic similarity between participants for an event related to the number of details recalled from that event and the number of intruding details (intrusions) that were present in the recall of that event. It is not strictly necessary that a greater number of recalled details should relate to higher topic similarity, but if participants have a tendency to recall similar latent themes, then recall of more information might allow for more precise estimation of topic proportions and higher similarity. Conversely, participants who mix in details from other events (intrusions) should have lower similarity to the rest of the group (unless everyone showed a tendency to have specific intrusions).

The number of intrusions per event was zero-inflated (70% of events recalled by participants had no intrusions) and thus we coded intrusions with a binary predictor (no intrusions: 0 or one or more intrusions: 1), and in a separate model, as a continuous variable excluding 0 values. Linear mixed models to predict topic similarity were fit for the normalized sum number of details recalled from each pair. In addition, separate models were fit to predict topic similarity from the binary categorization of intrusions and from the normalized sum of intrusions from the pair.

1 $${topicModelingSimilarity}\, \sim \,{su}{m}_{{detailsRecall}}+\left(1|{S}_{i}\right)+(1|{S}_{j})$$

2 $${topicModelingSimilarity}\, \sim \,{binar}{y}_{{intrusion}}+\left(1|{S}_{i}\right)+(1|{S}_{j})$$

3 $${topicModelingSimilarity}\, \sim \,{su}{m}_{{intrusion}}+\left(1|{S}_{i}\right)+(1|{S}_{j})$$

Next, a separate set of linear mixed models were fit to predict topic similarity from multivoxel intersubject pattern similarity from each ROI at both encoding and retrieval with a fully crossed random effect approach^31^ using lmer^32^ in R 4.4.1^33^. In these structures, each intersubject pattern similarity value in the model comes from a pair of participants *i* and *j*, where *i* ≠ *j*. Random effects are modeled for both the first and second participants in the pairing. Since the random effects are fully crossed, each participant appears in both the first random effect and the second random effect. However, this also leads to a data doubling in the model which is later corrected by discounting the degrees of freedom in the standard error of a term from the model and the degrees of freedom for the t-test and *p*-value calculations of those terms in line with Chen et al.^31^. This is done by dividing the number of observations by 2 and subtracting the number of fixed effects and using this value to replace the inflated degrees of freedom. The t-test statistics are recalculated using the fixed effects from the model and the updated standard error. Since topic similarity is calculated by a pair of participants, participant *i* and participant *j*, a linear mixed model was fit to predict topic similarity from the details recalled from participant *i* and the details recalled from participant *j* in line with Chen et al.^31^. Each participant contributes to both regressors for the details recalled for participant *i* and the details recalled from participant *j*, resulting in an identically estimated slope. To determine the unique effect of intersubject pattern similarity on topic similarity, we fit a separate set of models that included encoding intersubject pattern similarity and recalled details (for both participants) to predict topic similarity. While participants viewed all events during encoding, in the linear mixed models to predict encoding intersubject pattern similarity we only modelled events that are subsequently recalled by both participants in a pair because, if an event is not recalled, it is not possible to calculate topic similarity.

4 $${\!}{topicModelingSimilarity}\, \sim \,{encISPS}+{detailsRecal}{l}_{{s}_{i}}+{detailsRecal}{l}_{{s}_{j}}+\left(1|{S}_{i}\right)+(1|{S}_{j})$$

5 $${topicModelingSimilarity}\, \sim \,{recISPS}+{lengt}{h}_{{s}_{i}}+{lengt}{h}_{{s}_{j}}+\,\left(1|{S}_{i}\right)+(1|{S}_{j})$$

Also, when predicting topic similarity from recall intersubject pattern similarity, we included the number of TRs that elapsed as a participant described an event (i.e. the *length* of time they took recalling an event) as a regressor of no interest to account for the amount of data used to estimate multivoxel patterns. Significance was assessed at *p* < 0.05, FDR correcting for the number of predefined ROIs and all predictors were mean centered. The exploratory whole-brain analysis is presented unthresholded.

Next, we sought to examine whether there was a difference in the association between encoding intersubject pattern similarity versus recall intersubject pattern similarity with topic similarity. We included both pattern similarity measures in the same model to predict topic similarity and used a Wald Chi Square test to test the difference between the slopes. Significance was assessed at *p* < 0.05, FDR correcting for the number of predefined ROIs.

To determine whether the DMN had specificity in this relationship, we used a permutation test on the whole brain data. We calculated the difference in the mean slope estimates between the DMN and all other networks. We then produced a null distribution by randomly re-labelling each ROI and recalculating the differences 10,000 times. Significance was assessed by observing the proportion of times the true difference was greater or smaller than differences in the null distribution. Based on prior work^2,34^, we anticipated a positive difference in favor of the DMN and, thus, performed a one-sided test.

## Results

### Memory performance

Participants recalled an approximately two thirds of all events (9.25/14 for Barmaid and 9.66/14 for Bluestreets), recalling on average 6.25 details per events (86.75 details total for barmaid on average and 88.3 details total for Bluestreets on average). Recall was often exclusively focused on information that occurred in the described events with intrusions occurring in 30% of events recalled.

### Optimal number of topics

Pattern classifiers were able to accurately classify which event was being described in left out data for every value of K (chance = 1/14, accuracy range = 24%–56%). We observed that classifier accuracy increased with higher numbers of topics. The increase in accuracy showed diminishing returns between 19 and 31 topics for the two narratives. We then inspected the content of the topics identified by the LDA. We observed that beyond *K* = 19, the models produced degenerate topics characterized by semantically unrelated, low-frequency words (often appearing alphabetically ordered) with near uniform word distributions consistent with topics that were weakly constrained by the data. As such, *K* = 19 was selected as the optimal number of topics as it could significantly predict the event being described on left out data and was free from degenerate topics. Across the 100 iterations with differing random seeds, intraclass correlation was very high, ICC = 0.97.

### Similarity of topic patterns and scored details

We tested if recall similarity measured by topic similarity captured similar content as human raters. Linear mixed modeling revealed that event topic similarity was positively associated with the number of event details that were recalled (Supplemental Fig. 1), (t(3996) = 15.4, *p* < 0.0001, β = .06, S.E. = 0.002). Next, we observed that the binary presence of intrusions was significantly associated with topic similarity in a positive fashion, which was surprising (t(3996) = 2.36, *p* = 0.02, β = 0.01, S.E. = 0.004). Conversely, when looking at the number of intrusions as a continuous variable (excluding trials with 0 intrusions as this would create a zero inflated distribution), there was a significant negative relationship between the number of intrusions and topic similarity (t(1960) = −3.03, *p* = 0.003, β = -0.009, S.E. = 0.003). Given these contradictory findings, we examined whether these effects endured after accounting for the number of true event details (perhaps participants who provided many verifiable event details were also more likely to contribute intrusions). When re-running the linear mixed models to predict topic similarity from both the number of event details and the binary predictor of intrusions, the number of details remained significant (t(3995) = 25.7, *p* < 0.0001, β = 0.06, S.E. = 0.002), but the binary intrusions predictor was not (t(3995) = −1.05, *p* = 0.3, β = -0.004, S.E. = 0.003). When re-running the linear mixed models to predict topic similarity from both the number of event details and the number of intrusions, both the number of details (t(1959) = 16.7, *p* < 0.0001, β = 0.05, S.E. = 0.002) and the number of intrusions (t(1959) = −6.03, *p* < 0.0001, β = -0.02, S.E. = 0.002) remained significant. This demonstrates that the topics produced from the topic models are sensitive to the content of retrieval in an event-specific manner. When participants recall more true details and fewer intrusions, they show greater similarity.

### Relationship between topic similarity and intersubject pattern similarity

**Fig. 2:**
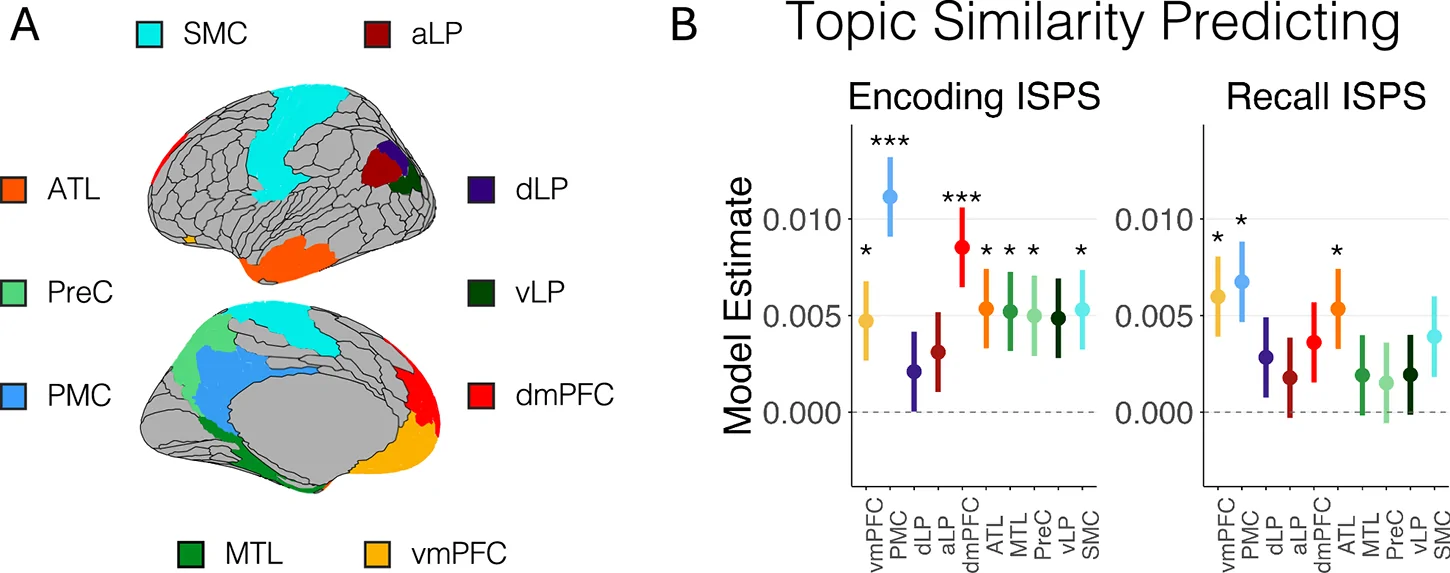
Topic similarity relates to intersubject pattern similarity (ISPS). Relationship between topic similarity and intersubject pattern similarity across regions of interest. **A** Depiction of ROIs on inflated brain. **B** Point plot showing the estimated model slopes for topic similarity when predicting intersubject pattern similarity at encoding (left) and recall (right). Error bars represent the standard error of the estimate. ATL, anterior temporal lobe; aLP, anterior lateral parietal cortex; dLP, dorsal lateral parietal cortex; dmPFC, dorsomedial prefrontal cortex; MTL, medial temporal lobe; PMC, posterior medial cortex; PreC, precuneus; SMC, somatomotor; vLP, ventral lateral parietal; vmPFC, ventromedial prefrontal cortex. *p < 0.05 FDR-correct; **p < 0.01 FDR-corrected; *** *p* < 0.001 FDR-corrected. *N* = 24.

Having observed that topic similarity is related to manual human scoring, we then asked whether topic similarity was associated with neural intersubject pattern similarity using linear mixed models. Given that enhanced intersubject correlations (i.e., temporally correlated timecourses between participants) during encoding have been associated with better memory performance^35–37^, we also included the number of details recalled in the same model to determine whether content similarity related to intersubject pattern similarity independent of just having better memory. We observed that topic similarity was significantly positively associated with encoding intersubject pattern similarity in most DMN regions, and also the control ROI (Fig. 2B) (PMC: t(3994) = 4.92, *p* < 0.0001; dmPFC: t(3994) = 3.84 *p* = 0.0006; ATL: t(3994) = 2.42, *p* = 0.03; PreC: t(3994) = 2.4, *p* = 0.03; vmPFC: t(3994) = 2.26, *p* = .04; MTL: t(3994) = 2.21, *p* = 0.04; SMC (control ROI): t(3994) = 2.56, *p* = 0.03; FDR-corrected). These results show that participants who had similar multivoxel patterns at encoding in cortical regions, especially PMC, tended to recall a similar collection of topics at retrieval. Test statistics and slopes for all regions are presented in Supplemental Table 2.

When looking at recall activity, topic similarity was positively associated with recall intersubject patterns in the PMC, vmPFC, and ATL (Fig. 2B) (PMC: t(3994) = 3.23, *p* = 0.012; vmPFC: t(3994) = 2.88, *p* = 0.02; ATL: t(3994) = 2.58, *p* = 0.033; FDR-corrected), but not other DMN cortical ROIs or the control ROI (dLP: t(3994) = 1.36, *p* = 0.29; dmPFC: t(3994) = 1.74, *p* = 0.16; MTL: t(3994) = 0.92, *p* = 0.44; PreC: t(3994) = 0.73, *p* = 0.47; vLP: t(3994) = 0.93, *p* = 0.44; aLP: t(3994) = 0.86, *p* = 0.44; SMC (control ROI): t(3994) = 1.87, *p* = 0.15, FDR-corrected). Test statistics and slopes for all regions are presented in Supplemental Table 3.

To examine whether intersubject pattern similarity varied in its relationship to topic similarity based on whether it was calculated at encoding or retrieval, we fit a linear mixed model to predict topic similarity from both encoding intersubject pattern similarity and recall intersubject pattern similarity, controlling for the number of recalled details. A Wald chi-squared test was used to determine whether the predictors significantly varied. After correcting for multiple comparisons, we did not see any significant differences between the predictors, but there was a trend showing that PMC intersubject pattern similarity was a marginally stronger predictor of topic similarity at encoding compared to recall (χ² = 7.05, p = .079).

### Effects beyond the DMN

Prior work has heavily implicated the DMN in representing event level information^2,34^, and interfacing with the hippocampus in service of memory encoding and retrieval^10,13^ and, thus, our main analysis focused on this network. We did not anticipate that representations in lower-level regions would play as significant a role in the memory similarity between participants. We performed an ROI-based whole brain analysis, repeating our above linear mixed models at every ROI to examine whether these effects were organized by standard intrinsic networks which we have previously identified (note that in our previous work the DMN and medial temporal network diverged and we present them as separate networks here)^8^. We used permutation testing to determine whether the DMN had specificity in its relationship between intersubject pattern similarity and topic similarity.

**Fig. 3:**
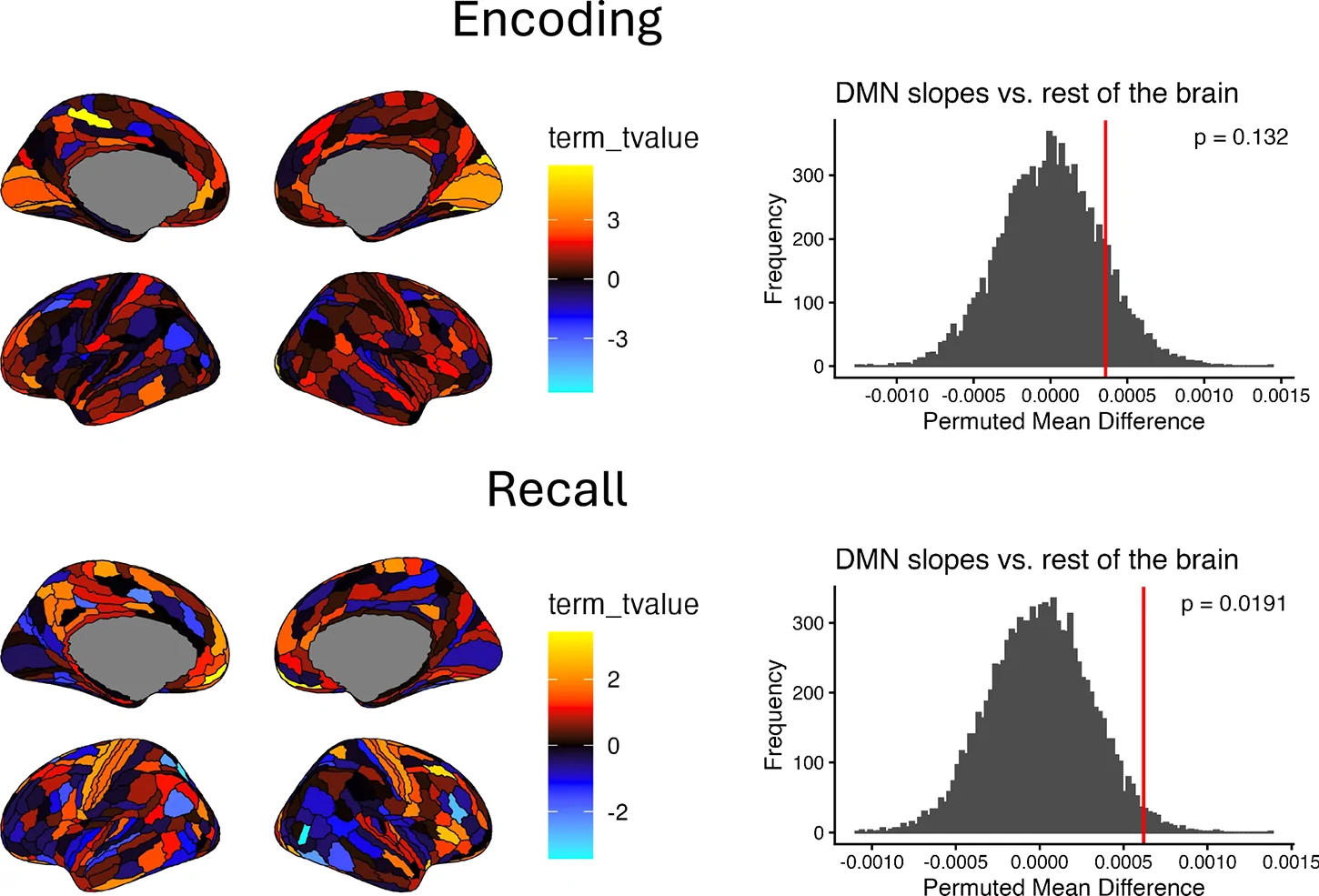
Relationship between topic similarity and intersubject pattern similarity across the cortex. Left: Unthresholded whole-brain ROI maps of t-values showing the relationship between intersubject pattern similarity and topic similarity using the HCP-MMP1.0 atlas^27^. Right: The null distribution of the difference between the slopes from the DMN and the rest of the brain created from 10,000 random shuffles of network labels of the ROIs present alongside the real observed difference, as the red vertical line. The *p*-value is the proportion of random shuffles that created a difference greater than the observed difference. *N* = 24.

At encoding, while the DMN had numerically higher slopes in the relationship between intersubject pattern similarity and topic similarity, we did not observe that the DMN showed specificity in this relationship (difference in mean slope between DMN and the rest of the brain = 0.0003, *p* = 0.13 (one-sided); Fig. 3). These results suggest that, at encoding, the effect is not unique to the DMN but may be expressed across regions in multiple networks. At recall, we observed that the DMN had an above average relationship between recall intersubject pattern similarity and topic similarity relative to the rest of the brain, and this specificity was significantly more reliable in the DMN (difference in mean slope between DMN and the rest of the brain = .00062, p = .019 (one-sided)).

## Discussion

People can often remember the same event in different ways. Here, we sought to examine what areas of the brain relate to the combination of features we recall. Using natural language processing tools, we quantified the similarity of memory content for naturalistic events between individuals. We found that the similarity of retrieved content tracked with the level of detail for those memories and was inversely related to the number of intrusions. Further, recall similarity was reliably associated with shared neural patterns across participants at encoding and retrieval and this was particularly true in posterior medial, ventromedial prefrontal, and anterior temporal cortex. Our hypotheses focused on the DMN, and whole-brain exploratory results revealed that these effects are especially strong in the DMN at retrieval, but not encoding. Together, these findings support the idea that the DMN represents latent event information both at encoding and retrieval.

Natural language processing and recently developed large language models are increasingly being explored to characterize memory retrieval and event cognition^17,38–43^. This work has shown that the abstract features generated from topic modeling or text embeddings can be used to segment narratives into discrete events similar to the way humans segment events^17,41,42^ and have linked changes in semantic content with neural state changes in DMN regions^44^. Further, these models are beginning to be used to score recall accuracy showing concordance with human raters^45,46^ as well as to examine the diversity of information retrieved^47^. We trained a topic model on the recall transcripts generated by participants in the study to calculate a set of latent topics at the group level. We observed that when participants were able to generate more details, they also tended to show greater similarity in the pattern of topics used to describe a given event. However, and importantly, when participants generated intrusions (details from another event), we observed lower similarity in the pattern of topics used to describe the event. These results provide peace of mind that the topic models trained here are capturing the latent features present when participants recall the same events.

Previous work has demonstrated that multivariate patterns within cortical regions of the DMN are event-specific and shift at event boundaries during encoding^48,49^. These same patterns are reinstated at retrieval^2,3^, and the magnitude of reinstatement is proportional to the amount of hippocampal boundary activity observed when these patterns switch^48^. These DMN regions are thought to represent the ongoing event model of the current situation^5,11,50^. That is to say, these regions are thought to integrate the current ongoing experience with prior event knowledge to form an abstracted representation that allows for interpretation and predictions of incoming information^51^. Indeed, when events share abstracted event information, multivariate patterns in these areas are more likely to generalize^19,34,52^. During encoding, interaction between the hippocampus and DMN at event boundaries seem to allow for the long term storage of these abstracted representations allowing for successful subsequent retrieval^10,13,53^. This degree of abstraction fits with topographic network analysis of the brain that has shown the DMN sits at the apex of a cortical hierarchy^54,55^ receiving abstracted external information from intermediary networks^8^. Further, as people attend and encode incoming information, changes in semantic content coincide well with multivariate changes in DMN regions^44^. Our findings, showing a relationship between abstracted topics and multivariate patterns in the DMN fit well with the notion that the DMN is capturing abstracted or compressible higher-order information in service of event cognition^56^. Collectively, this evidence points to the DMN’s role in combining incoming information with prior semantic knowledge to represent the ongoing interpretation of events, ultimately impacting the content recalled at retrieval.

Remarkably, multivariate patterns within the DMN at encoding and recall are shared between people^2,3^. Our work extends this to show that the degree of overlap in these patterns is associated with the content of subsequent retrieval. Previous work has shown that when two people watch the same stimulus, but have different interpretations, they will have more distinct patterns and dynamics in these regions compared to when they have the same interpretation^15,16,57^. Our findings are strongly concordant with these previous observations and capitalize on the memory representations generated by the participants to identify subject to subject and event to event variations in recall similarity. Our results extend on this previous work showing that the spatial patterns established during encoding in DMN regions (such as the PMC, vmPFC and ATL) are indicative of the subsequent memory representations that will be formed and retrieved. These effects are even more impressive given that they are found even after accounting for the detailedness of memory representations. Importantly, however, whole-brain ROI analysis revealed widespread contributions. This may highlight that subsequent memory representations are influenced by multiple factors including higher-order context, attentional contributions, and mid to lower-order sensory signals.

We, additionally, observed relationships between recall similarity and recall intersubject pattern similarity. These findings dovetail well with prior work^16^. Zadbood et al.^16^ observed that participants had greater pattern similarity when recalling a narrative with a similar interpretation compared to recalling it with a different interpretation. This effect was found in ventromedial prefrontal cortex, posterior medial cortex and lateral temporal cortex—regions that overlap strongly with our findings. This effect is particularly impressive given that the amount of fMRI data available from participants is less than the amount of data at encoding since people tend to temporally compress events when recalling them^58^. These findings together point to representations in the DMN carrying event-level information at both encoding and retrieval, suggesting a greater role in ongoing experience beyond just internally directed thought or attentional states^59^.

We had hypothesized that recall similarity and recall intersubject pattern similarity relationships would be specific to regions with strong coupling to the hippocampus such as medial temporal and DMN regions. When examining the mean slopes across the different networks in the brain, we observed that the DMN showed higher slopes than expected compared to other networks in the brain confirming the specificity of higher order cortex in representing abstract recalled content.

### Limitations

One challenge with natural language processing models is the multiple model parameters that can be explored. Additionally, the training of topic models can result in slightly different weightings and groupings each time due to the stochastic nature of the word by document decomposition. To account for these challenges, we identified the optimal number of topics that would allow a pattern classifier to classify events while minimizing redundant, degenerate topics. We also averaged across multiple iterations of model training runs to ensure the results were not merely based on a random seed. Additionally, in our study, we relied on verbal free recall to estimate memory representation similarity. Given that people tend to have different levels of verbosity, we may be able to better represent latent themes present in some participants’ memory representations compared to others who speak more reservedly. This limitation is typical in free recall of naturalistic or autobiographical event studies, even when experimenter probing is provided to request more information^60^. Future studies could ask specific probing questions about memories and interpretations surrounding aspects of events that may be subserved by different regions or subnetworks of the DMN^8,61–63^. For example, one could specifically ask about emotional tones or themes, the meaning of social interactions or sarcasm, or perceptual details. These focused questions could provide deeper insight into where individuals converge or diverge in their recollections and interpretations. This approach could also address a key limitation of topic modeling: the difficulty in interpreting the resulting latent topics. These topics often lack a clear semantic categorization or label. Asking targeted questions in future experiments could help link neural representations with specific types of content.

### Conclusions

In conclusion, the present study demonstrates that activity patterns within the DMN during encoding predict the content people subsequently retrieve. These findings reinforce the role of the DMN in maintaining event model representations that reflect our ongoing understanding of an event and our eventual memory representations for that event. These findings also highlight the utility of topic modeling in providing insight in characterizing memory representations.

## Supplementary information

Transparent Peer Review file

Supplemental Information

## Data Availability

The data used to create figures is available via GitHub (https://github.com/ajbarn/recall_similarity_ISPS).

## Code availability

The code used to create figures is available via GitHub (https://github.com/ajbarn/recall_similarity_ISPS).
