# Supplementary material for: Natural language processing captures memory content associated with shared neural patterns at encoding and retrieval
Source: Commun Psychol. 2026 Jun 4;4:124. doi: 10.1038/s44271-026-00481-0 (PMC13547101; doi:10.1038/s44271-026-00481-0)
Supplement: Supplementary file 1 [file 44271_2026_481_MOESM1_ESM.pdf]

nature portfolio

Peer Review File

Natural language processing captures memory content associated with shared neural patterns at encoding and retrieval

Corresponding Author: Dr Alexander Barnett

This file contains all editorial decision letters in order by version, followed by all author rebuttals in order by version.

Version 0:

Decision Letter:

** Please ensure you delete the link to your author homepage in this e-mail if you wish to forward it to your coauthors **

Dear Dr Barnett,

Thank you for your patience during the peer-review process. Your manuscript titled "Natural language processing captures memory content associated with shared neural patterns at encoding" has now been seen by 4 reviewers, and I include their comments at the end of this message. They find your work of interest but raised some important points. We are interested in the possibility of publishing your study in Communications Psychology, but would like to consider your responses to these concerns and assess a revised manuscript before we make a final decision on publication.

We therefore invite you to revise and resubmit your manuscript, along with a point-by-point response to the reviewers. Please highlight all changes in the manuscript text file.

Editorially, we consider the most important points to address to be clarifying the study's conceptual novelty relative to prior research, providing fuller methodological transparency (including event definition, recall scoring, topic modeling parameters, and participant/task details), and strengthening justification of analytic choices. The revision should also include more complete reporting of behavioral and model statistics, control ROI results, and either a whole-brain analysis or more convincing evidence for DMN specificity.

As you revise the manuscript in response to these issues, please also implement all requests in the attached Mandatory Revision Requests document. All requirements listed in this document need to be fully met, or the work will be returned to you for further revisions without peer review. This workflow is in place to increase the likelihood that the paper will be accepted for publication. It reduces the number of rounds of revision (and review) and ensures that the reviewers vet a version of the article that is compliant with journal policies. If you have any questions regarding the required revisions, please contact the journal prior to resubmission to avoid a negative outcome.

We are committed to providing a fair and constructive peer-review process. Please don't hesitate to contact us if you wish to discuss the revision in more detail.

Please submit the following items:

- Revised manuscript
- Point-by-point response to the referees' comments
- Mandatory Revision Requests Table (attached).
- Cover letter (as a separate document)
- Nature Research Reporting Summary (for

research Articles, Stage 2 Registered Reports, and Resources only)

via this link: Link Redacted .

** This url links to your confidential home page and associated information about manuscripts you may have submitted or are reviewing for us. If you wish to forward this email to co-authors, please delete the link to your homepage first **

We hope to receive your revised paper within 8 weeks; please let us know if you aren't able to submit it within this time so that we can discuss how best to proceed. If we don't hear from you, and the revision process takes significantly longer, we may close your file. In this event, we will still be happy to reconsider your paper at a later date, provided it still presents a significant contribution to the literature at that stage.

We look forward to seeing the revised manuscript and thank you for the opportunity to review your work.

Best regards,

Troby Lui, on behalf of

Jesse Rissman

Troby Lui, PhD
Associate Editor
Communications Psychology

Jesse Rissman, PhD
Editorial Board Member
Communications Psychology
orcid.org/0000-0001-8889-5539

REVIEWER EXPERTISE:
Reviewer #1 (human memory, fMRI, NLP, multivariate analysis)
Reviewer #2 (human memory, fMRI, NLP, multivariate analysis)
Reviewer #3 (human memory, fMRI, NLP, multivariate analysis)
Reviewer #4 (human memory, fMRI, NLP, multivariate analysis)

REVIEWER REPORTS:

Reviewer #1 (Remarks to the Author):

This manuscript examines how shared neural patterns in the Default Mode Network (DMN) relate to similarity in memory recall. Twenty-four participants viewed two cartoon movies and freely recalled them during fMRI. Recall transcripts were analyzed with topic modeling to quantify memory similarity across participants, which was then used to predict intersubject pattern similarity in DMN regions. The results show that higher topic similarity between participants was positively related to the number of verifiable details recalled and negatively related to intrusions. Critically, topic similarity was significantly associated with ISPS during encoding across multiple DMN regions, particularly the posterior medial cortex, and to a lesser extent during recall. The authors argue that these findings indicate shared neural patterns at encoding can predict the similarity of later recall, while also demonstrating the value of natural language processing for quantifying memory content. Overall, although the results generally appear consistent with existing literature and the authors' hypotheses, several conceptual and methodological concerns remain that, if addressed, would strengthen the manuscript.

1. The novelty and significance of the study are somewhat unclear. The extensive literature on DMN function, together with studies on intersubject differences in neural responses to the same stimuli already cited in the manuscript, raises the question of how much this work advances our understanding of the DMN. Furthermore, the application of natural language processing techniques, such as topic modeling, has been widely used in memory research for several years, as the authors themselves acknowledge.

2. Several key procedural details are missing. Although the authors note that more detailed methods can be found in their earlier publication (Barnett et al., 2024), including additional methodological descriptions in the current manuscript would help readers better understand and follow the study.

- It should be made clearer in the abstract and introduction that this is a reanalysis of a previously published dataset.
- Providing a bit more information about the movie stimuli could be helpful.
- The recall procedure also requires clarification: did recall occur after participants watched both movies, or after each

individual movie? From the task instructions, it appears there were four functional runs with alternating movie-watching and recall phases, but this is not clearly stated in either the "Dataset" or "MRI acquisition" sections.
- The description of recall scoring is also vague and should specify exactly what the transcripts were "scored" for.
- The definition of "events" is unclear. The manuscript does not specify the criteria used to define event boundaries, how many raters were involved, or whether any steps were taken to ensure consistency across raters. Additionally, it does not report how many events occurred per movie or their average duration.

3. Several analytical choices require clearer justification and explanation.

- One of the main research questions in this manuscript is "to examine what areas of the brain relate to the combination of features we recall." To address this question and support the claim that their effects are DMN-specific, the authors should perform a whole-brain analysis. Using the somatomotor cortex as a single control region does not provide a convincing case for DMN specificity.
- Why did the authors use topic modeling rather than sentence embeddings generated via pretrained large language models? Such embeddings can be easily generated and do not require the parameter selection necessary for topic modeling. Would the authors observe the same results using LLM-generated embeddings?
- The parameter sweep approach (5–55 topics) is difficult to justify, particularly if topic similarity values were averaged across models with different numbers of topics. A more typical approach would be to first select the number of topics based on an optimization criterion, such as perplexity (ideally using cross-validation), and then apply the chosen model to the entire dataset. Relatedly, the variance resulting from different random seeds or numbers of topics should be reported.
- The regression model used to extract voxel activity per event needs more detail, including whether models were generated separately for each run and whether additional regressors such as motion parameters were included.
- It would be helpful to explain the rationale for testing the relationship between the number of details/intrusions and topic similarity earlier in the Methods section, rather than introducing it only in the Results.
- More detail is also needed on the linear mixed models. For example, was movie or functional run included as a random effect? Was the "number of TRs" regressor included for both participants in the pair? It would be clearer to show the model itself in formula form (e.g., similarity~S1detail+S2detail+(1|S1)+(1|S2)).
- Please clarify whether the reported FDR correction was applied across ROIs or across any other factors.

4. Some of the statistical analysis results appear incomplete or unclear.

- Basic descriptive statistics for the behavioral data are missing and should be provided to help readers interpret the results. For example, the authors should report the average number of details recalled per event and per movie, the number and percentage of intrusions, and the percentage of events recalled versus forgotten.
- The reporting of mixed-model statistics appears incomplete. Currently, only t values and p values are provided, without parameter estimates, standard errors, or confidence intervals. It is also unclear how the degrees of freedom (e.g., df = 3993) were determined.
- It is unclear whether any likelihood ratio tests (e.g., full model vs. null model) or other analyses were performed to assess the significance of the predictors.
- In the "similarity of topic patterns and scored details" section, the models testing the relationship between topic similarity and the number of details recalled or intrusions each report a single statistical test. However, the Methods describe each mixed model as including two separate regressors for the number of details or intrusions (i.e., for subject i and subject j in the pair). If that is the case, why is only one statistic reported?

5. Other minor comments and miscellaneous errors

- On page 4, line 82, the results reported are incomplete. The text introduces analyses of detail counts, intrusions, and recall ISPS, but only encoding ISPS is described. The outcomes for details, intrusions, and recall ISPS should be reported to match what was introduced.
- Figure 1 would be improved by including illustrative examples of latent topics, such as representative word lists or sample topic distributions for one event. This would make the concept of "topic similarity" more concrete to readers.
- The second paragraph of the Discussion needs a more in-depth interpretation of the relationship between the number of details, intrusions, and topic similarity. At present this relationship is described only briefly and could be elaborated to show what it reveals about memory content and variability.
- On page 6, line 158, "we divided the recall transcript into separate 'segments' based on…". The word "segments" is missing.
- On page 7, lines 196 and 200, The citation style is incorrect. It should be either "in line with a prior study (G. Chen et al., 2017)" or "in line with G. Chen et al. (2017)," but not "in line with (G. Chen et al., 2017)."
- On page 7, line 205, the text should read "a set of models that included both 'topic similarity' and recalled details." not "included both 'ISPS' and recalled details."

Reviewer #2 (Remarks to the Author):

Overview

The authors asked participants to watch and recall in detail two cartoon movies while undergoing fMRI. The main finding is that greater inter-subject pattern similarity in the DMN during encoding predicts greater similarity in recall (as characterized by topic models). Overall this is a solid and interesting study. I have some relatively minor suggestions, below.

Minor comments (presented in order of appearance in the text):

1. When I read the Intersubject pattern similarity analysis description in the methods section (p. 5), I was initially surprised that the authors limited the analysis to DMN regions. I had gotten the impression that they were *testing* whether DMN shows the similarity effect more strongly than other regions. In re-reading the abstract and introduction I can see that the authors were describing their approach accurately– but it might help other readers to add another sentence or two to make it explicit that they are specifically limiting the analysis to DMN regions (as opposed to *comparing* DMN to other regions/networks).

2. The correct HyperTools reference (p. 6) is Heusser et al. (2018), not Pedregosa et al. (2011).

3. In Figure 2A and B (p. 9) it would be nice to (a) use shared y-axes between the two panels and (b) either show individual data points (I realize there are many of them) or overlay the line plots on 2-D histograms (if showing individual data points is too messy). The two results in that figure are interesting, and clearly (from the stats reported on p. 9) they are statistically reliable– but it's difficult to get a sense from the figures alone how the individual observations are distributed.

4. On p. 9 (bottom paragraph) the authors mention replicating the "topic similarity vs. encoding ISPS" analysis for a control ROI. However, I don't see stats for that control ROI (or associated figures) reported in the text. Similarly, in Fig. 3 (p. 10) it would be nice to also include the control ROI (in both panels) for comparison.

5. One of the most fascinating "results" (sort of?) from the paper is the implication (p. 10) that ISPS at *encoding* better predicts recall topic similarity than ISPS at *recall*. But this is left implicit– the authors comment that "on the other hand, … recall activity…[is associated with topic similarity] only at uncorrected levels [in a subset of DMN regions]". It would be helpful to present an explicit statistical test comparing the strength of ISPS-recall topic similarity associations for activity recorded during encoding vs. recall. A challenge (which the authors note on p. 12) is that participants have higher motion during recall, and therefore interpreting a weaker effect during recall requires deconfounding a true neural difference from motion artifacts. But perhaps it would be possible to regress out the effects of motion? In any case, even with caveats about interpretation, it would strengthen the story to include a direct comparison of the strengths of the associations between recall topic similarity and encoding vs. recall ISPS.

Reviewer #3 (Remarks to the Author):

I co-reviewed this manuscript with one of the reviewers who provided the listed reports. This is part of the Communications Psychology initiative to facilitate training in peer review and to provide appropriate recognition for Early Career Researchers who co-review manuscripts.

Reviewer #4 (Remarks to the Author):

Review of Kim
The current manuscript describes a novel analysis of behavioral and functional imaging data collected from a group of 24 individuals, towards the goal of exploring correlated event patterns in the default mode network (DMN) while watching a cartoon that overlap with the similarity in recalled events during that cartoon. The central premise of this work is the DMN represents event models, and that correlated patterns of activity across individuals during shared experiences are possible evidence of shared representation of high-level event features. The authors test this idea by combining NLP topic modeling based on recall transcriptions with a derived neural measure, "ISPS" (intersubject pattern similarity), which quantifies the correlation of mean activity for discrete events during two separate cartoons. The strength of the manuscript is driven by 2 main findings. First, greater similarity in recalled content was associated with stronger shared activation patterns at encoding. Second, topic similarity across participants increased as a function of the number of details recalled, supporting the hypothesis that similarity carries meaningful information. I find the manuscript to be well designed and motivated, and have only one general comment, and a number of more minor comments towards a future revision.

One of the principal strengths and challenges for these data is suggestion that the DMN carries meaningful information from encoding forward to the recall of the narrative events. The data here successfully challenges many dominant views of DMNs as largely agnostic to information processing and more driven by factors like "state", awareness, or attention. Nonetheless, the data presented here is nonetheless not conclusive on their argument, and alternative hypotheses are not rigorously entertained.

Relatedly, more clarity on what is concretely being represented by their analysis, beyond intrasubject similarity, would solidify the findings. Participants watched a 16 minute cartoon, and a topic modeling approach identified 5-55 topics that might show a common occurrence in free recall between participants. But we are not given information on what kind of cartoon was seen, what kinds of topics were identified, so it is difficult to link the data with the inferences made. While previously explained in Barnett et al. 2024, more details on the movie stimuli would be very useful, including a description of the characters, events, or contexts that comprise "event features".

More details on the participant pool are necessary (age, # of M/F, compensation, IRB approval).

MRI procedure/acquisition details on the recall period are unclear. What were the run lengths for the recall runs? Did scanner noise interfere with transcription? There are no details about the MRI acquisition for this part of the paradigm, even though the fMRI data during the recall period comprises the main focus of the analysis.

Ln 143: The authors estimate the mean activity across an event, and then calculated the similarity in the activity across events; how many events are used to generate the Pearson’s correlation? How long are these events?

More broadly, the manuscript needs to make clear that the ISPS analysis was conducted during recall, not encoding.

This is very subjective, but Figure 1 (bottom) implies that event boundaries are a strong component of the data, but these are never explicitly modeled in the current data. Furthermore, it could be made clearer that the authors are averaging across discrete periods of the timeseries, and that TR-to-TR fluctuations (i.e., typical FC) are not at play here. The title specifies “Model mean event activity”, but this is ambiguous. Authors may wish to adjust the timeseries depiction to de-emphasize this.

Signed,
Simon Davis
Indiana University

* TRANSPARENT PEER REVIEW: Communications Psychology uses a transparent peer review system. This means that we publish the editorial decision letters including Reviewers' comments to the authors and the author rebuttal letters online as a supplementary peer review file. However, on author request, confidential information and data can be removed from the published reviewer reports and rebuttal letters prior to publication. If your manuscript has been previously reviewed at another journal, those Reviewers' comments would not form part of the published peer review file.

** Visit Nature Research's author and referees' website at www.nature.com/authors for information about policies, services and author benefits**

Communications Psychology is committed to improving transparency in authorship. As part of our efforts in this direction, we are now requesting that all authors identified as ‘corresponding author’ create and link their Open Researcher and Contributor Identifier (ORCID) with their account on the Manuscript Tracking System prior to acceptance. ORCID helps the scientific community achieve unambiguous attribution of all scholarly contributions. You can create and link your ORCID from the home page of the Manuscript Tracking System by clicking on ‘Modify my Springer Nature account’ and following the instructions in the link below. Please also inform all co-authors that they can add their ORCIDs to their accounts and that they must do so prior to acceptance.
https://www.springernature.com/gp/researchers/orcid/orcid-for-nature-research

For more information please visit http://www.springernature.com/orcid

If you experience problems in linking your ORCID, please contact the Platform Support Helpdesk.

Version 1:

Decision Letter:

** Please ensure you delete the link to your author homepage in this e-mail if you wish to forward it to your coauthors **

Dear Dr Barnett,

Your manuscript titled "Natural language processing captures memory content associated with shared neural patterns at encoding and retrieval" has now been seen by our reviewers, whose comments appear below. In light of their advice I am delighted to say that we are happy, in principle, to publish a suitably revised version in Communications Psychology.

We therefore invite you to revise your paper one last time to address the remaining concerns of our reviewers and a list of editorial requests. At the same time we ask that you edit your manuscript to comply with our format requirements and to maximise the accessibility and therefore the impact of your work.

EDITORIAL REQUESTS:

Please review our specific editorial comments and requests regarding your manuscript in the attached "Editorial Requests Table". Please outline your response to each request in the right hand column. Please upload the completed table with your manuscript files as a Related Manuscript file.

If you have any questions or concerns about any of our requests, please do not hesitate to contact me.

SUBMISSION INFORMATION:

In order to accept your paper, we require the files listed here https://www.nature.com/documents/commsj-file-checklist.pdf .

OPEN ACCESS:

Communications Psychology is a fully open access journal. Articles are made freely accessible on publication. For further information about article processing charges, open access funding, and advice and support from Nature Research, please visit https://www.nature.com/commspsychol/open-access

At acceptance, you will be provided with instructions for completing the open access licence agreement on behalf of all authors. This grants us the necessary permissions to publish your paper. Additionally, you will be asked to declare that all required third party permissions have been obtained, and to provide billing information in order to pay the article-processing charge (APC).

* TRANSPARENT PEER REVIEW: Communications Psychology uses a transparent peer review system. On author request, confidential information and data can be removed from the published reviewer reports and rebuttal letters prior to publication. If you are concerned about the release of confidential data, please let us know specifically what information you would like to have removed. Please note that we cannot incorporate redactions for any other reasons.

* CODE AVAILABILITY: All Communications Psychology manuscripts must include a section titled "Code Availability" at the end of the methods section. We require that the custom analysis code supporting your conclusions is made available in a publicly accessible repository at this stage; please choose a repository that generates a digital object identifier (DOI) for the code; the link to the repository and the DOI must be included in the Code Availability statement. Publication as Supplementary Information will not suffice.

* DATA AVAILABILITY:
All Communications Psychology manuscripts must include a section titled "Data Availability" at the end of the Methods section. More information on this policy, is available in the Editorial Requests Table and at http://www.nature.com/authors/policies/data/data-availability-statements-data-citations.pdf.

Please use the following link to submit the above items:
Link Redacted
** This url links to your confidential home page and associated information about manuscripts you may have submitted or be reviewing for us. If you wish to forward this email to co-authors, please delete the link to your homepage first **

We hope to hear from you within two weeks; please let us know if you need more time.

Best regards,

Troby Lui, on behalf of

Jesse Rissman

Troby Lui, PhD
Associate Editor
Communications Psychology

Jesse Rissman, PhD
Editorial Board Member
Communications Psychology
orcid.org/0000-0001-8889-5539

REVIEWERS' COMMENTS:

Reviewer #1 (Remarks to the Author):

The authors have addressed some of the previous concerns and added a few methodological details. However, several issues remain that should be clarified or corrected.

1. In the last paragraph of the Introduction (p. 4), the authors revised the text to indicate that the current study reanalyzes a previously published dataset. However, the revision seems to unnecessarily remove most of the information describing that prior study. Even if the dataset has been published previously, the introduction should still provide at least a brief description of the original study (e.g., its design).

2. In the section “Effects beyond the DMN” (pp. 14-15), the reported statistical tests are inconsistent. Some regions are reported with one-sided tests (e.g., DMN, MTL), while some are reported with two-sided tests (e.g., AUD). It is unclear why different testing directions are used across regions. Unless there is a clear a priori justification for using one-sided tests for specific networks, the statistical test should be applied consistently.

3. On page 8, the manuscript states that an optimal number of topics (K) was selected, but the text still includes the sentence, “The resulting topic similarity values were averaged across the range of topic numbers”, which is confusing. Does this mean that the authors still average across different values of K even after selecting the optimal K?
In addition, the plots showing model performance as a function of the number of topics should be reported as a supplementary figure. The manuscript also does not report the variance associated with different random seeds (this could be shown as error bars in the supplementary figure).

4. Remaining minor issues in the manuscript:

-Page 7, line 207: The sentence “we divided the recall transcript into separate based on…” is missing the word “segments.” This issue was pointed out previously but remains unaddressed.

-Pages 9-10, the model description still contains an error that was pointed out before but not addressed. The manuscript states: “we fit a set of models that included encoding intersubject pattern similarity and recalled details … to predict intersubject pattern similarity.” The predictor should be 'topic similarity'.

-There is an inconsistency in the reported degrees of freedom. On pages 13–14, the revised manuscript reports t(3994), but Supplemental Table 2 and Table 3 report df = 3993.

-Page 5, line 144: The sentence “Thus, run lengths varied between participants from (108s to 1016s; mean = 688s)” is ungrammatical and should be revised.

-The response to Reviewer 2, Comment 5 appears to be truncated in the response letter. The text ends with “Beyond the simple”.

Reviewer #2 (Remarks to the Author):

The authors have done a nice job with their revision. I have just a few minor suggestions:

1. The HyperTools ref is here (note: 2018, not 2021): https://www.jmlr.org/papers/v18/17-434.html

2. I suggest including the scatterplots showing topic similarity vs. intrusions and details recalled in supplemental materials. And given the shapes of those distributions, I'd suggest toning down the associated claims (using your Bayesian/t-distribution approach) accordingly. They look a bit messy, so that should be acknowledged in some form.

3. Your response to my 5th "concern" (listed under R2) appears to have been cut off (ends at "Beyond the simple" with no rest of the sentence). But, extrapolating from what I can see: if there is no interaction between topic similarity and phase, that should be made clear in the manuscript and to report those stats.

Reviewer #3 (Remarks to the Author):

I co-reviewed this manuscript with one of the reviewers who provided the listed reports. This is part of the Communications Psychology initiative to facilitate training in peer review and to provide appropriate recognition for Early Career Researchers who co-review manuscripts.

Reviewer #4 (Remarks to the Author):

The revised manuscript represents a thorough revision of the analysis and presentation of the authors application of NLP tools to an fMRI encoding and recall dataset. The authors have addressed all extant concerns, including more details on the MRI acquisition and task paradigm, greater detail on what comprises an "event" in their analysis, a small clarification on the role of the DMN, and more minor modifications requested throughout. The more refined version of this manuscript is a pleasure to read.

** Visit Nature Research's author and referees' website at www.nature.com/authors for information about policies, services and author benefits**

Open Access This Peer Review File is licensed under a Creative Commons Attribution 4.0 International License, which permits use, sharing, adaptation, distribution and reproduction in any medium or format, as long as you give appropriate credit to the original author(s) and the source, provide a link to the Creative Commons license, and indicate if changes were made.
In cases where reviewers are anonymous, credit should be given to 'Anonymous Referee' and the source.
The images or other third party material in this Peer Review File are included in the article's Creative Commons license, unless indicated otherwise in a credit line to the material. If material is not included in the article's Creative Commons license and your intended use is not permitted by statutory regulation or exceeds the permitted use, you will need to obtain permission directly from the copyright holder.
To view a copy of this license, visit https://creativecommons.org/licenses/by/4.0/

REVIEWER REPORTS:

Reviewer #1 (Remarks to the Author):

This manuscript examines how shared neural patterns in the Default Mode Network (DMN) relate to similarity in memory recall. Twenty-four participants viewed two cartoon movies and freely recalled them during fMRI. Recall transcripts were analyzed with topic modeling to quantify memory similarity across participants, which was then used to predict intersubject pattern similarity in DMN regions. The results show that higher topic similarity between participants was positively related to the number of verifiable details recalled and negatively related to intrusions. Critically, topic similarity was significantly associated with ISPS during encoding across multiple DMN regions, particularly the posterior medial cortex, and to a lesser extent during recall. The authors argue that these findings indicate shared neural patterns at encoding can predict the similarity of later recall, while also demonstrating the value of natural language processing for quantifying memory content.
Overall, although the results generally appear consistent with existing literature and the authors' hypotheses, several conceptual and methodological concerns remain that, if addressed, would strengthen the manuscript.

1. The novelty and significance of the study are somewhat unclear. The extensive literature on DMN function, together with studies on intersubject differences in neural responses to the same stimuli already cited in the manuscript, raises the question of how much this work advances our understanding of the DMN. Furthermore, the application of natural language processing techniques, such as topic modeling, has been widely used in memory research for several years, as the authors themselves acknowledge.

We have worked to clarify the novelty in the manuscript. Broadly, we are the first to show that shared multivariate patterns during narrative encoding are later associated with greater similarity of recall content. This is well motivated by previous literature, but is novel. People who have similar background knowledge of a narrative tend to have similar neural representations at encoding (Zadbood et al 2022) and people who represent an event similarly to the group in terms of neural patterns also tend to be able to recall that event at a later time. Here, we connect these ideas to show that not only is the memory retrievable, but the content of what is being recalled, measured by topic similarity, is more overlapping.

Building from these findings, one might expect that people who have similar DMN event representations at encoding may go on to recall similar details at retrieval. To our knowledge, no study to date has examined this relationship. Further, while previous studies have shown that people with similar interpretations at recall have similar DMN events patterns, these studies compared participants in discrete groups by experimentally manipulating their interpretations of events (Yeshurun et al., 2017; Zadbood et al., 2022). These manipulations involved either selectively providing or withholding information to bias the meaning of events (e.g. one group

gets to see the end of a narrative which reframes how earlier events are viewed). However, such approaches risk oversimplifying the multifaceted nature of narrative memory. For example, one pair of subjects may share many aspects of their recall, another pair may share a few, and another pair may have opposing views of an event. Recent advances in natural language processing models have provided powerful tools for analyzing the language people use during recall (Nguyen et al., 2019). These models can uncover latent themes in spoken or written recollections, allowing researchers to identify both shared and unique aspects of remembered content (Heusser et al., 2021). By examining which combinations of latent themes are common across pairs of participants, this approach enables a more nuanced assessment of the similarity in their memory representations.

2. Several key procedural details are missing. Although the authors note that more detailed methods can be found in their earlier publication (Barnett et al., 2024), including additional methodological descriptions in the current manuscript would help readers better understand and follow the study.

Thank you for providing us with a clear guide on how to make the methodology clearer for our readers.

- It should be made clearer in the abstract and introduction that this is a reanalysis of a previously published dataset.

We have now clarified in the abstract and the introduction that this analysis was done on a preexisting dataset

Abstract:
Here, we examined whether memory similarity correlates with intersubject spatial patterns in the DMN using a pre-existing dataset.

Introduction:
To do so, we re-analyzed data from Barnett et al (2024).

- Providing a bit more information about the movie stimuli could be helpful.

We have provided additional information on the movie stimuli in the methods:

These custom-made cartoons were made using Plotagon Studio (https://www.plotagon.com/desktop/). The movies depicted a continuous narrative, one following a stereotypical crime scene investigation style story and the other depicting a medieval fantasy style story. Each movie consisted of 14 events (mean 72s in length) which were defined by shifts in location, time, and character composition. These events were confirmed and identified by a sample of 62 online participants from Barnett et al (2024) who were instructed to watch one of the videos and press the spacebar when they felt that "one meaningful event had ended and

another had begun" in line with prior work in the field (Ben-Yakov & Henson, 2018; Sargent et al., 2013). The resulting timecourse of button presses from each participant was combined to form a distribution which was modelled with a kernel density estimate. Peaks in the density confirmed the intended event boundaries designed in the stimuli (and identified an additional boundary in each stimulus). These were used as "event boundaries" in Barnett et al (2024). Here, an "event" is defined as the time between one event boundary and the next.

- The recall procedure also requires clarification: did recall occur after participants watched both movies, or after each individual movie? From the task instructions, it appears there were four functional runs with alternating movie-watching and recall phases, but this is not clearly stated in either the "Dataset" or "MRI acquisition" sections.

Thank you for the suggestion. We have now clarified that participants watched and recalled the videos in turn in the MRI acquisition section.

Participants viewed and verbally recalled each video in turn during fMRI.

- The description of recall scoring is also vague and should specify exactly what the transcripts were "scored" for.

We have provided more details in the methods of the scoring.

Audio recall for each participant was transcribed and scored. First, each participant's transcript was divided into events based on the content being described by trained raters. For example, if the participant said "In the next scene, Arlene and Muller talked outside in the burning village …" then this and the text that immediately follows would be classified as event 13—the penultimate event of one of the movies. This event would be classified as finished when the participant changed to describing a different event (e.g. "Next we cut to the ballroom where we see the Count waiting for Muller's return"). Two trained raters then scored each event for the number of details recalled. Details were defined as "a unique occurrence, observation, or thought, typically expressed as a grammatical clause. Additional information in the clause was scored separately" as defined by Levine et al. (2002). For example, "they wanted to talk privately…" is one detail and "…so they went inside the bar" is another detail. Only details that were verifiably true according to the events depicted in the movies were considered. We then calculated the sum of these verifiable details recalled for each event. In some cases, participants would recall a detail from another event during the recounting of an event. These details were classified as intrusions. For additional information on scoring and labeling procedures, see ref Barnett et al., 2024.

- The definition of "events" is unclear. The manuscript does not specify the criteria used to define event boundaries, how many raters were involved, or whether any steps were taken to ensure consistency across raters. Additionally, it does not report how many events occurred per movie or their average duration.

We have provided additional information on the events and their definition in the methods:

Each movie consisted of 14 events (mean 72s in length) which were defined by shifts in location, time, and character composition. These events were confirmed and identified by a sample of 62 online participants from Barnett et al (2024) who were instructed to watch one of the videos and press the spacebar when they felt that "one meaningful event had ended and another had begun" in line with prior work in the field (Ben-Yakov & Henson, 2018; Sargent et al., 2013). The resulting timecourse of button presses from each participant was combined to form a distribution which was modelled with a kernel density estimate. Peaks in the density confirmed the intended event boundaries designed in the stimuli (and identified an additional boundary in each stimulus). These were used as "event boundaries" in Barnett et al (2024). Here, an "event" is defined as the time between one event boundary and the next.

3. Several analytical choices require clearer justification and explanation.

- One of the main research questions in this manuscript is "to examine what areas of the brain relate to the combination of features we recall." To address this question and support the claim that their effects are DMN-specific, the authors should perform a whole-brain analysis. Using the somatomotor cortex as a single control region does not provide a convincing case for DMN specificity.

This is a really good point made by the reviewer. The choice to focus on the DMN was made for multiple reasons.

1. Its inherent connections with the hippocampus and the role this plays in and retrieval encoding of narrative event information
2. Its previous shared multivoxel patterns during encoding and recall of narrative information

We have added in a supplementary whole brain analysis in addition to our targeted ROI approach to examine DMN specificity.

Methods:

To examine multivoxel patterns, we present both a predefined ROI approach in which we assess the contributions of regions of the DMN alongside an exploratory whole-brain approach. For the whole brain, we used 360 parcels from the HCP-MMP1.0 atlas (Glasser et al., 2016).

Results

Effects beyond the DMN

Prior work has heavily implicated the DMN in representing event level information, and interfacing with the hippocampus in service of memory encoding and retrieval and, thus, our main analysis focused on this network. We did not anticipate that representations in lower-level regions would play as significant a role in the memory similarity between participants. We performed an ROI-based whole brain analysis to examine these effects organized by standard intrinsic networks which we have previously identified (note that in our previous work the DMN and medial temporal network diverged and we present them as separate networks here) (Barnett

et al., 2021). To determine whether the DMN had specificity in this relationship, we used a permutation test. We calculated the difference in the mean slope estimates between the DMN and all other networks. We then produced a null distribution by randomly re-labelling each ROI and recalculating the differences 10,000 times. Significance was assessed by observing the proportion of times the true difference was greater or smaller than differences in the null distribution.

We observed that the DMN had a stronger relationship between encoding ISPS and topic similarity compared to the rest of the brain, p = .039 (one-sided). Notably, we observed that the medial temporal and auditory networks also had relatively high relationships between encoding ISPS and topic similarity. We, thus, explored these networks finding that they also showed higher slopes compared to the rest of the brain (MTL p = .035 (one-sided); AUD p = .01 (two-sided)). These results suggest that, at encoding, the effect is not unique to the DMN but is expressed across regions in multiple networks, including DMN, medial temporal, and auditory regions. At recall, however, we observed that the DMN had a stronger relationship between recall ISPS and topic similarity relative to the rest of the brain, p = .0002 (one-sided).

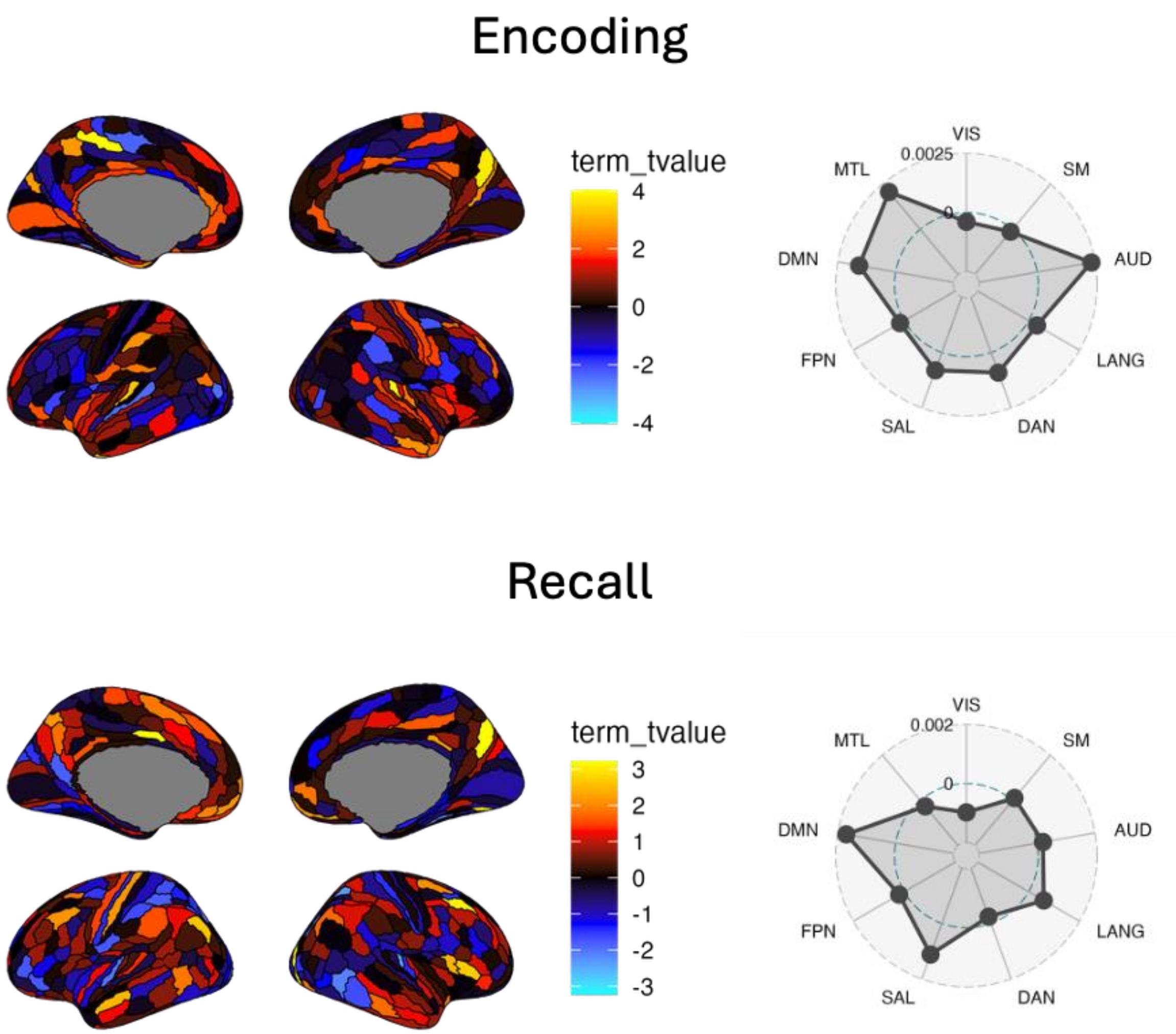

Figure 3. Unthresholded whole-brain ROI maps of t-values showing the relationship between ISPS and topic similarity using the HCP-MMP1.0 atlas (Glasser et al., 2016) on the left and radar plots of the mean slope for each network on the right.

- Why did the authors use topic modeling rather than sentence embeddings generated via pretrained large language models? Such embeddings can be easily generated and do not require the parameter selection necessary for topic modeling. Would the authors observe the same results using LLM-generated embeddings?

Topic modeling was selected as it is able to learn the latent structure that exists within a narrative. For example, one latent topic might be biased to the words “arlene”, “gonzalo”, “street”, “ship” which would be diagnostic of one event compared to a topic that is sensitive to “arlene”, “hector”, “street”, “ship”. In this sense, the co-occurrence of characters and locations is relevant to the topic model, whereas such relationships would likely not be relevant features in a LLM trained on a large corpus.

- The parameter sweep approach (5–55 topics) is difficult to justify, particularly if topic similarity values were averaged across models with different numbers of topics. A more typical approach would be to first select the number of topics based on an optimization criterion, such as perplexity (ideally using cross-validation), and then apply the chosen model to the entire dataset. Relatedly, the variance resulting from different random seeds or numbers of topics should be reported.

We thank the reviewer for their recommendation. Rather than simply averaging over a range of topics, we have now implemented an optimization criteria for selecting the K number of topics. Rather than using perplexity (see below), we opted to optimize K by selecting the K that maximizes the predictability of “event label” of left out data from a trained pattern classifier. This was balanced against whether the topics created were coherent.

Methods:

When training a topic model, certain parameters can affect the model, like the number of topics (K). Given that we didn't have a strong prior regarding the number of topics, we performed a parameter sweep from 5 - 100 topics spaced by 5. To optimize the number of topics, at each topic K, we split the data into a training dataset (80% of the data) and a testing data set (20%). A topic model was fit to the training dataset and used to transform both the training and testing dataset into their constituent event topic vectors. A pattern classifier was then trained on the training dataset to predict event labels based on event topic vectors and tested on the left out testing dataset using multinomial logistic regression with L2 penalty. This was done for each movie separately and repeated 30 times at each topic K. Model accuracy was plotted against topic number to identify inflection points in model performance where increasing K resulted in diminishing returns. Next, we examined the latent topics identified from the LDA to evaluate their interpretability and their redundancy. After selecting the optimal K, we calculated topic similarity. The fitting of the topic model will vary based on a random seed, resulting in slightly different models each time. To ensure that our estimates of topic similarity are not sporadic, we

also fit the model 100 times using a new random seed the optimal K number of topics and averaged the similarity to bootstrap our findings and used the average topic similarity. The 'batch' learning method was used, and all other parameters were kept as default. The resulting topic similarity values were averaged across the range of topic numbers.

Results

Optimal number of topics

We observed that classifier accuracy increased with higher numbers of topics. The increase in accuracy showed diminishing returns between 17 and 31 topics for the two narratives. We then inspected the content of the topics identified by the LDA. We observed that beyond K = 19, the model produced degenerate topics characterized by semantically unrelated, low-frequency words (often appearing alphabetically ordered) with near uniform word distributions consistent with topics that were weakly constrained by the data. As such, K = 19 was selected as the optimal number of topics.

Movie 1

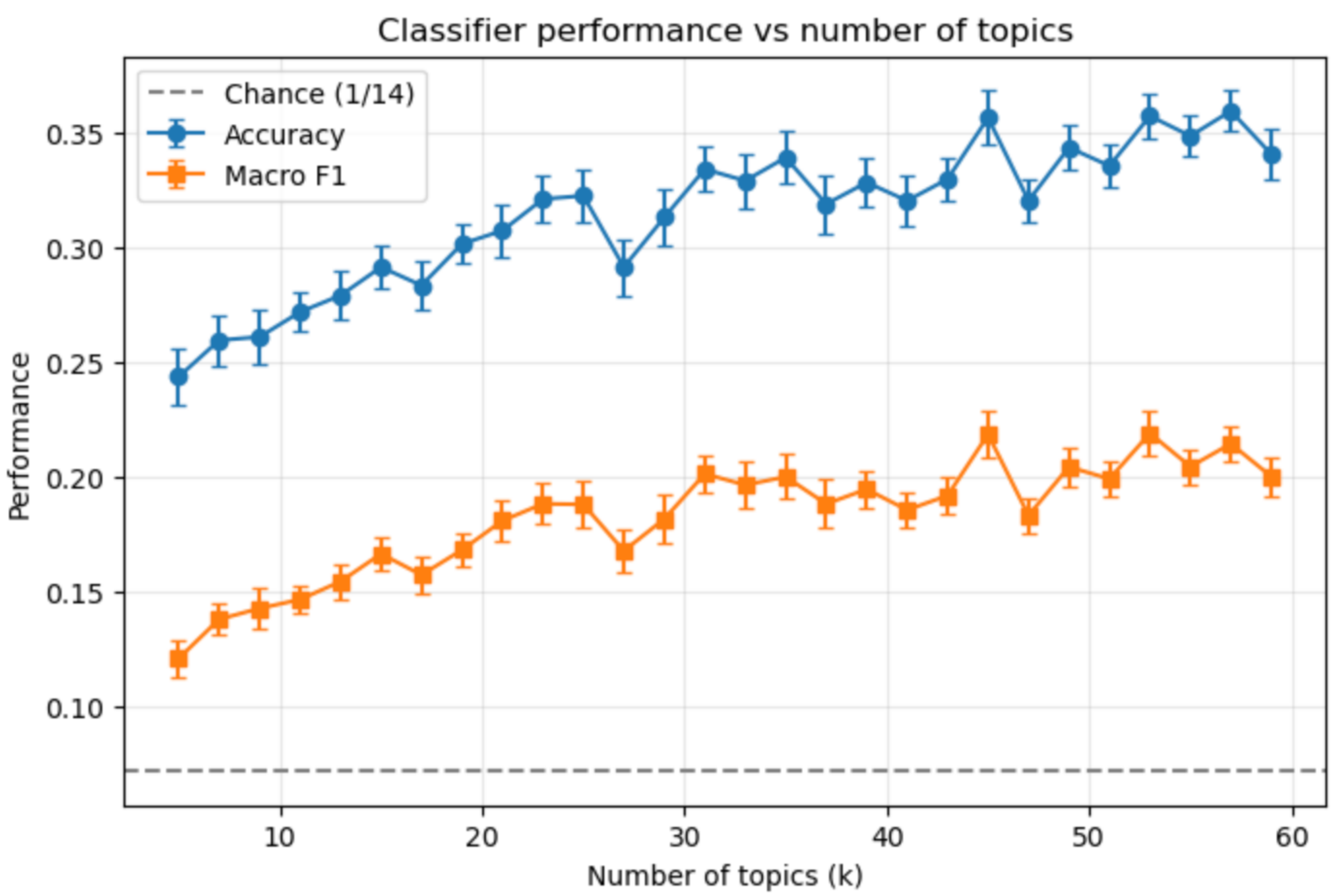

Movie 2

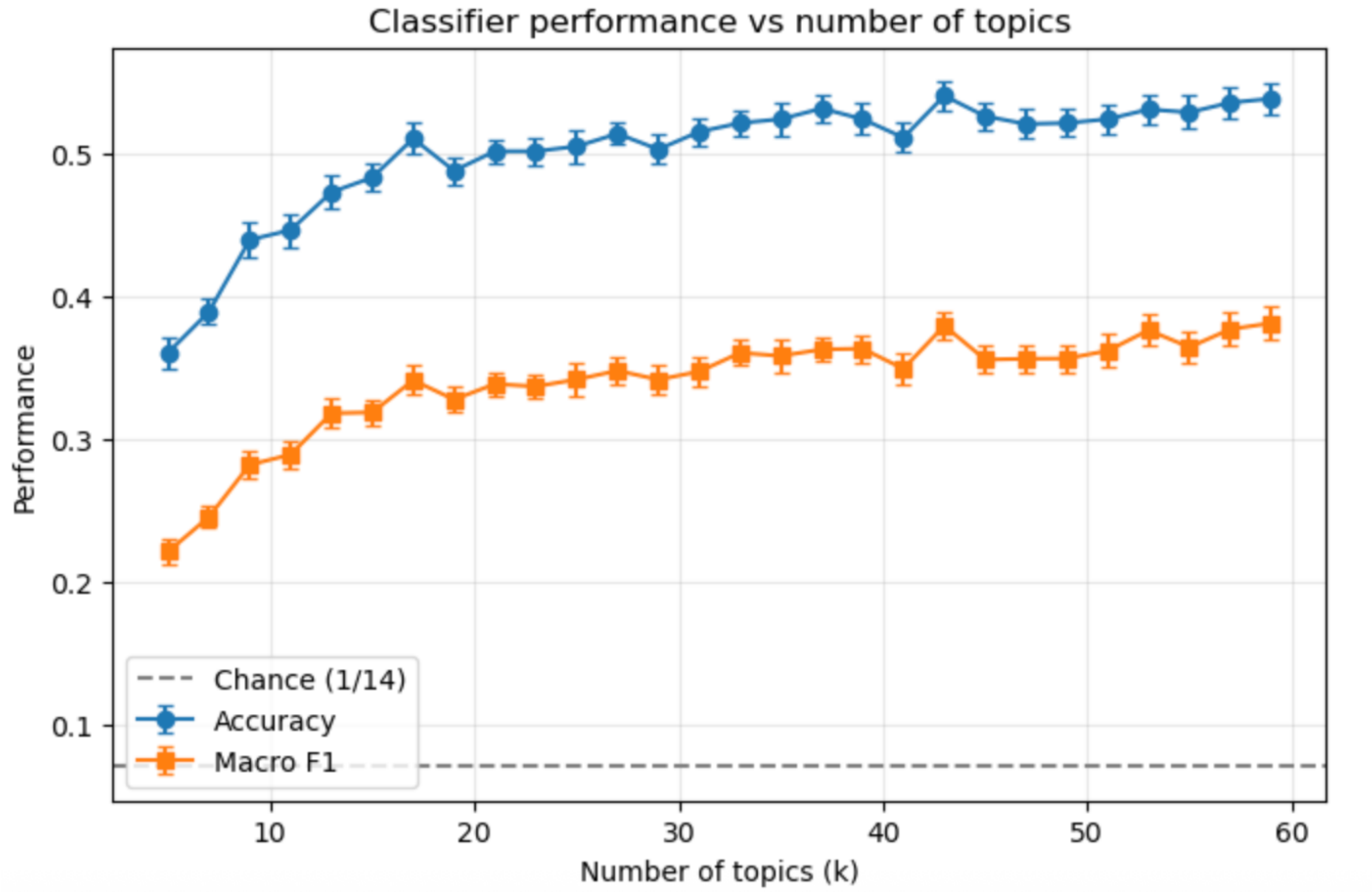

Perplexity in our dataset increased monotonically as the number of topics increased, indicating that additional topics reduced the model's ability to predict unseen data. According to perplexity, the optimal k = 2 for both narratives. This solution is insufficient to capture the event level structure of the data. Thus, we opted to identify the topic number K that would best capture the event level structure via pattern classifier, but also maintain topic coherence.

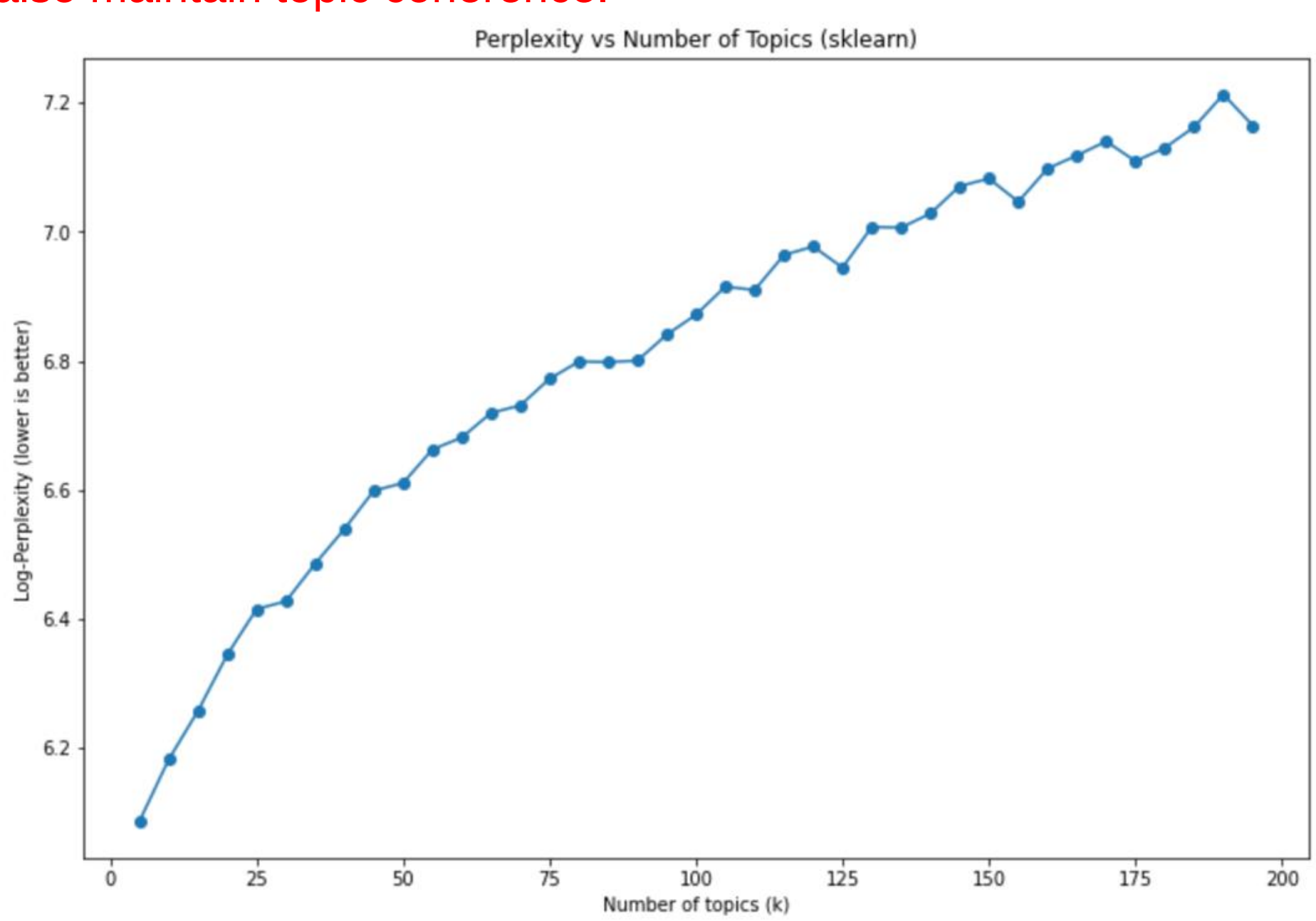

- The regression model used to extract voxel activity per event needs more detail, including whether models were generated separately for each run and whether additional regressors such as motion parameters were included.

Additional regressors such as motion parameters were not included in the model estimating mean voxel activity per event because motion was regressed out of the data in a prior step.

Regressors of no interest including six-degree motion parameters and motion outliers (framewise displacement > 0.5 mm, DVARS > 3) were regressed out of the data and the data was high-pass filtered to remove frequencies below .001Hz all in one step using tproject (Cox & Hyde, 1997) in nipype (Gorgolewski et al., 2011).

- It would be helpful to explain the rationale for testing the relationship between the number of details/intrusions and topic similarity earlier in the Methods section, rather than introducing it only in the Results.

Thank you for the suggestion, we have incorporated the rationale in the methods section now.

Statistical Analyses
First, we examined how the topic similarity between participants for an event related to the number of details recalled from that event and the number of intruding details (intrusions) that were present in the recall of that event. It is not strictly necessary that a greater number of recalled details should relate to higher topic similarity, but if participants have a tendency to recall similar latent themes, then recall of more information might allow for more precise estimation of topic proportions and higher similarity. Conversely, participants who mix in details from other events (intrusions) should have lower similarity to the rest of the group (unless everyone showed a tendency to have specific intrusions).

- More detail is also needed on the linear mixed models. For example, was movie or functional run included as a random effect? Was the "number of TRs" regressor included for both participants in the pair? It would be clearer to show the model itself in formula form (e.g., similarity~S1detail+S2detail+(1|S1)+(1|S2)).

The number of TRs regressor was included for both participants. We have adopted the formula into the manuscript to make the modeling clearer.

$$encISPS \sim topicModelingSimilarity + detailsRecall_{s1} + detailsRecall_{s2} + (1|S1) + (1|S2)$$

$$recISPS \sim topicModelingSimilarity + length_{s1} + length_{s2} + (1|S1) + (1|S2)$$

- Please clarify whether the reported FDR correction was applied across ROIs or across any other factors.

We have now added this detail.

Significance was assessed at $p < .05$, FDR correcting for the number of pre-defined ROIs

4. Some of the statistical analysis results appear incomplete or unclear.

- Basic descriptive statistics for the behavioral data are missing and should be provided to help readers interpret the results. For example, the authors should report the average number of details recalled per event and per movie, the number and percentage of intrusions, and the percentage of events recalled versus forgotten.

We have now added these details.

Memory performance

Participants recalled an approximately two thirds of all events (9.25/14 for Barmaid and 9.66/14 for Bluestreets), recalling on average 6.25 details per events (86.75 details total for barmaid on average and 88.3 details total for Bluestreets on average). Recall was often exclusively focused on information that occurred in the described events with intrusions occurring in only 30% of events recalled.

- The reporting of mixed-model statistics appears incomplete. Currently, only t values and p values are provided, without parameter estimates, standard errors, or confidence intervals. It is also unclear how the degrees of freedom (e.g., df = 3993) were determined.

We have added the parameter estimates and standard errors into supplemental table 2 for complete reporting.

- It is unclear whether any likelihood ratio tests (e.g., full model vs. null model) or other analyses were performed to assess the significance of the predictors.

We have clarified the approach to assess significance of the predictors in the methods section:

Next, a separate set of linear mixed models were fit to predict multivoxel ISPS from topic similarity for each ROI at both encoding and retrieval with a fully crossed random effect approach(G. Chen et al., 2017) using lmer (Bates et al., 2015) in R 4.4.1 (R Core Team, 2021). In this structure, each ISPS value in the model comes from a pair of participants i and j, where $i \neq j$. Random effects are modeled for both the first and second participants in the pairing. Since

the random effects are fully crossed, each participant appears in both the first random effect and the second random effect. However, this also leads to a data doubling in the model which is later corrected by discounting the degrees of freedom in the standard error of a term from the model and the degrees of freedom for the t-test and p-value calculations of those terms in line with G. Chen et al. 2017 by dividing the number of observations by 2 and subtracting the number of fixed effects. The t-test statistics are recalculated using the fixed effects from the model and the updated standard error. Since topic similarity is calculated by a pair of participants, participant i and participant j, a linear mixed model was fit to predict topic similarity from the details recalled from participant i and the details recalled from participant j in line with G. Chen et al. 2017. To determine whether ISPS was driven more by topic similarity or recalled details, we fit a separate set of models that included both ISPS and recalled details (for both participants) in the model to predict ISPS. While participants viewed all events during encoding, in the linear mixed models to predict encoding ISPS only modelled events that are subsequently recalled by both pairs of participants because if an event is not recalled, it is not possible to calculate topic similarity.

- In the “similarity of topic patterns and scored details” section, the models testing the relationship between topic similarity and the number of details recalled or intrusions each report a single statistical test. However, the Methods describe each mixed model as including two separate regressors for the number of details or intrusions (i.e., for subject i and subject j in the pair). If that is the case, why is only one statistic reported?

There are indeed separate fixed regressors for subject i and subject j for details and intrusions. Every subject is represented each in subject i and subject j (and correspondingly, every subject is represented in the random effects of subject i and subject j). This results in 1. the separate regressors being completely identical and 2. a data doubling causing a doubling of the degrees of freedom. This means that the parameter estimates and variances are identical, but come from doubled data. Thus, we adjust the degrees of freedom and only report the effects once. We have updated the methods to explicitly state this.

Since topic similarity is calculated by a pair of participants, participant i and participant j, a linear mixed model was fit to predict topic similarity from the details recalled from participant i and the details recalled from participant j in line with G. Chen et al. 2017. Each participant contributes to both regressors for the details recalled for participant i and the details recalled from participant j, resulting in an identically estimated slope.

5. Other minor comments and miscellaneous errors

- On page 4, line 82, the results reported are incomplete. The text introduces analyses of detail counts, intrusions, and recall ISPS, but only encoding ISPS is described. The outcomes for details, intrusions, and recall ISPS should be reported to match what was introduced.

The results overall have changed and we would update this in the final paragraph of the introduction but we have been guided by the journal to remove all mention of methods and results in this final paragraph.

- Figure 1 would be improved by including illustrative examples of latent topics, such as representative word lists or sample topic distributions for one event. This would make the concept of “topic similarity” more concrete to readers.

This is a great idea. We have included a few examples of the topic activations for 3 events and the words the contribute to the peak representative topics of the events.

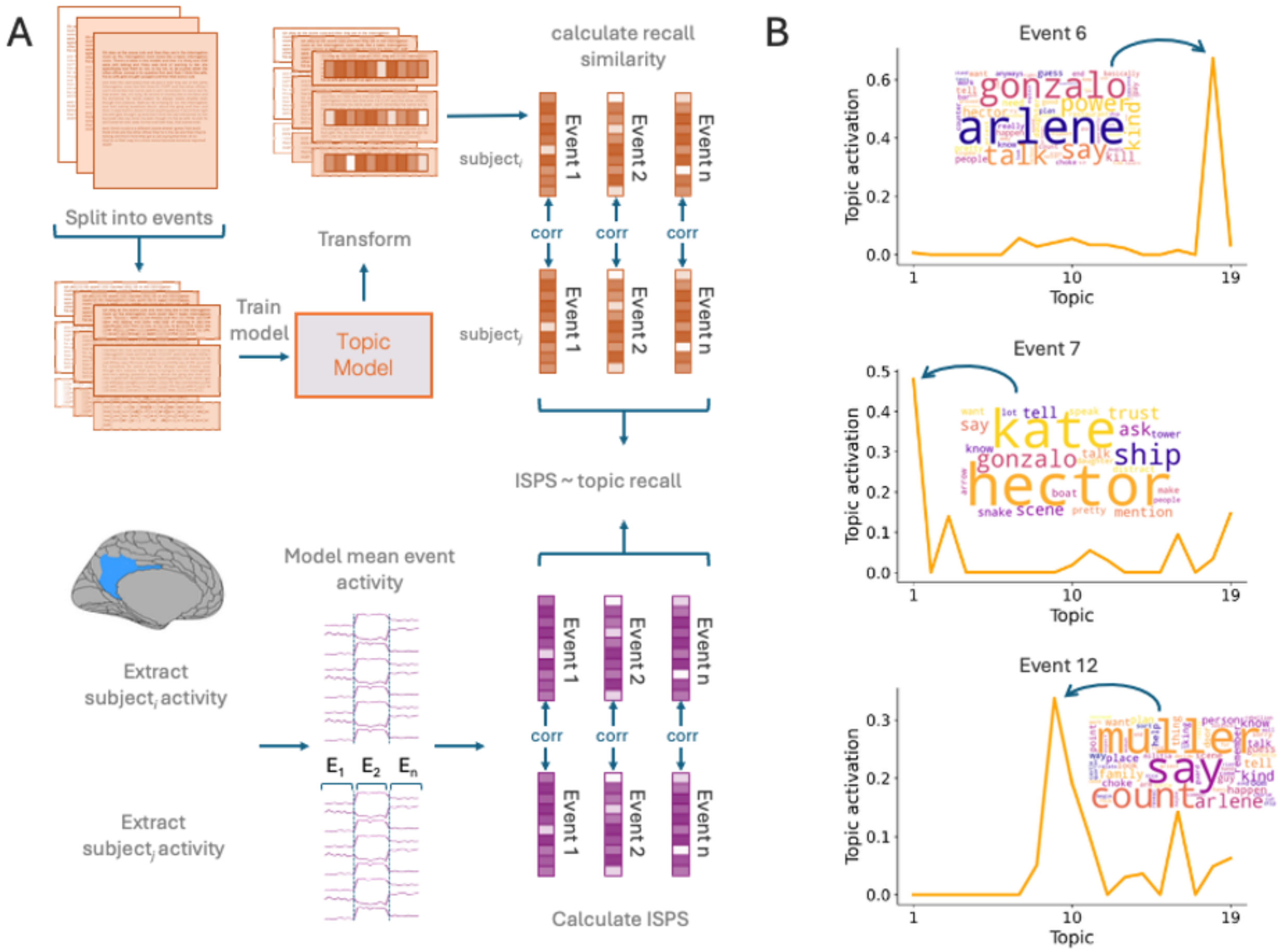

Figure 1. A. *Overview of analysis pipeline. TOP: The preprocessed recall transcripts for each participant were divided into separate documents for each event. The collection of documents was then used to train a topic model to identify latent topics. Each event from each participant was then transformed into a vector of topic proportions that reveal the combination of topics present in the recalled event using the trained topic model. To calculate topic similarity, we correlated the vector of topic proportions from matching events between participants and Fisher z-transformed the correlation. BOTTOM: To examine intersubject pattern similarity (ISPS), multivoxel activity was extracted from each ROI and was modeled to find the mean multivoxel pattern for each event and each participant. We then correlated these multivoxel patterns for matching events between pairs of participants and Fisher-z transformed the correlation to get ISPS.* ***B.*** *Plots showing the average topic embeddings for three example events along with word*

clouds highlighting the top words that contribute to those latent topics (larger words contribute more to topic activation). Of note, topics often consisted of present or mentioned characters along with spatial location or topics of conversation (e.g. Hector, Kate, Ship, for event 7 that depicts those characters on a boat).

- The second paragraph of the Discussion needs a more in-depth interpretation of the relationship between the number of details, intrusions, and topic similarity. At present this relationship is described only briefly and could be elaborated to show what it reveals about memory content and variability.

We would not assert that these findings are revealing much about memory content and its variability. The fact that latent patterns are positively correlated with numbers of details and negatively with intrusions may suggest that the participants are not making systematic intrusions at a rate high enough to be picked up by latent factors in a topic model, but these results are more for grounding the validity of the topic modeling approach and is not optimally designed to uncover principles of memory content or variability. For example, work from Sheldon et al (2024) was able to use topic modeling on autobiographical recall in an aging context to examine the variety of topics recalled during the recall of a specific cued event. This study examined a wide range of memories and employed a topic model that had a wide range of diverse topics to provide insight into the approach older and younger adults taking to recounting autobiographical events. Topic model in the current manuscript was more limited to searching as a tool for quantifying similarity.

- On page 6, line 158, “we divided the recall transcript into separate ‘segments’ based on…”. The word “segments” is missing.
- On page 7, lines 196 and 200, The citation style is incorrect. It should be either “in line with a prior study (G. Chen et al., 2017)” or “in line with G. Chen et al. (2017),” but not “in line with (G. Chen et al., 2017).”

Corrected. Thank you.

- On page 7, line 205, the text should read “a set of models that included both ‘topic similarity’ and recalled details.” not “included both ‘ISPS’ and recalled details.”

Reviewer #2 (Remarks to the Author):

Overview

The authors asked participants to watch and recall in detail two cartoon movies while undergoing fMRI. The main finding is that greater inter-subject pattern similarity in the DMN during encoding predicts greater similarity in recall (as characterized by topic models). Overall this is a solid and interesting study. I have some relatively minor suggestions, below.

Thank you for your kind words.

Minor comments (presented in order of appearance in the text):

1. When I read the Intersubject pattern similarity analysis description in the methods section (p. 5), I was initially surprised that the authors limited the analysis to DMN regions. I had gotten the impression that they were *testing* whether DMN shows the similarity effect more strongly than other regions. In re-reading the abstract and introduction I can see that the authors were describing their approach accurately– but it might help other readers to add another sentence or two to make it explicit that they are specifically limiting the analysis to DMN regions (as opposed to *comparing* DMN to other regions/networks).

Thank you for this suggestion. It was also brought up by another reviewer that our manuscript produced the expectation of exploring the DMN vs. other networks. We have 1. Bolstered our hypothesis driven approach and 2. Provided a whole brain, unthresholded view of these effects to examine the specificity of the DMN.

Introduction:

Building from these findings, one might expect that people who have similar DMN event representations at encoding may go on to recall similar details at retrieval. To our knowledge, no study to date has examined this relationship. Further, while previous studies have shown that people with similar interpretations at recall have similar DMN events patterns, these studies compared participants in discrete groups by experimentally manipulating their interpretations of events (Yeshurun et al., 2017; Zadbood et al., 2022).

Results:

Effects beyond the DMN

Prior work has heavily implicated the DMN in representing event level information, and interfacing with the hippocampus in service of memory encoding and retrieval and, thus, our main analysis focused on this network. We did not anticipate that representations in lower-level regions would play as significant a role in the memory similarity between participants. We performed an ROI-based whole brain analysis to examine these effects organized by standard intrinsic networks which we have previously identified (note that in our previous work the DMN and medial temporal network diverged and we present them as separate networks here) (Barnett et al., 2021). To determine whether the DMN had specificity in this relationship, we used a permutation test. We calculated the difference in the mean slope estimates between the DMN and all other networks. We then produced a null distribution by randomly re-labelling each ROI and recalculating the differences 10,000 times. Significance was assessed by observing the proportion of times the true difference was greater or smaller than differences in the null distribution.

We observed that the DMN a stronger relationship between encoding ISPS and topic similarity compared to the rest of the brain, $p = .039$ (one-sided). Notably, we observed that the

medial temporal and auditory networks also had relatively high relationships between encoding ISPS and topic similarity. We, thus, explored these networks finding that they also showed higher slopes compared to the rest of the brain (MTL p = .035 (one-sided); AUD p = .01 (two-sided)). These results suggest that, at encoding, the effect is not unique to the DMN but is expressed across regions in multiple networks, including DMN, medial temporal, and auditory regions. At recall, however, we observed that the DMN had a stronger relationship between recall ISPS and topic similarity relative to the rest of the brain, p = .0002 (one-sided).

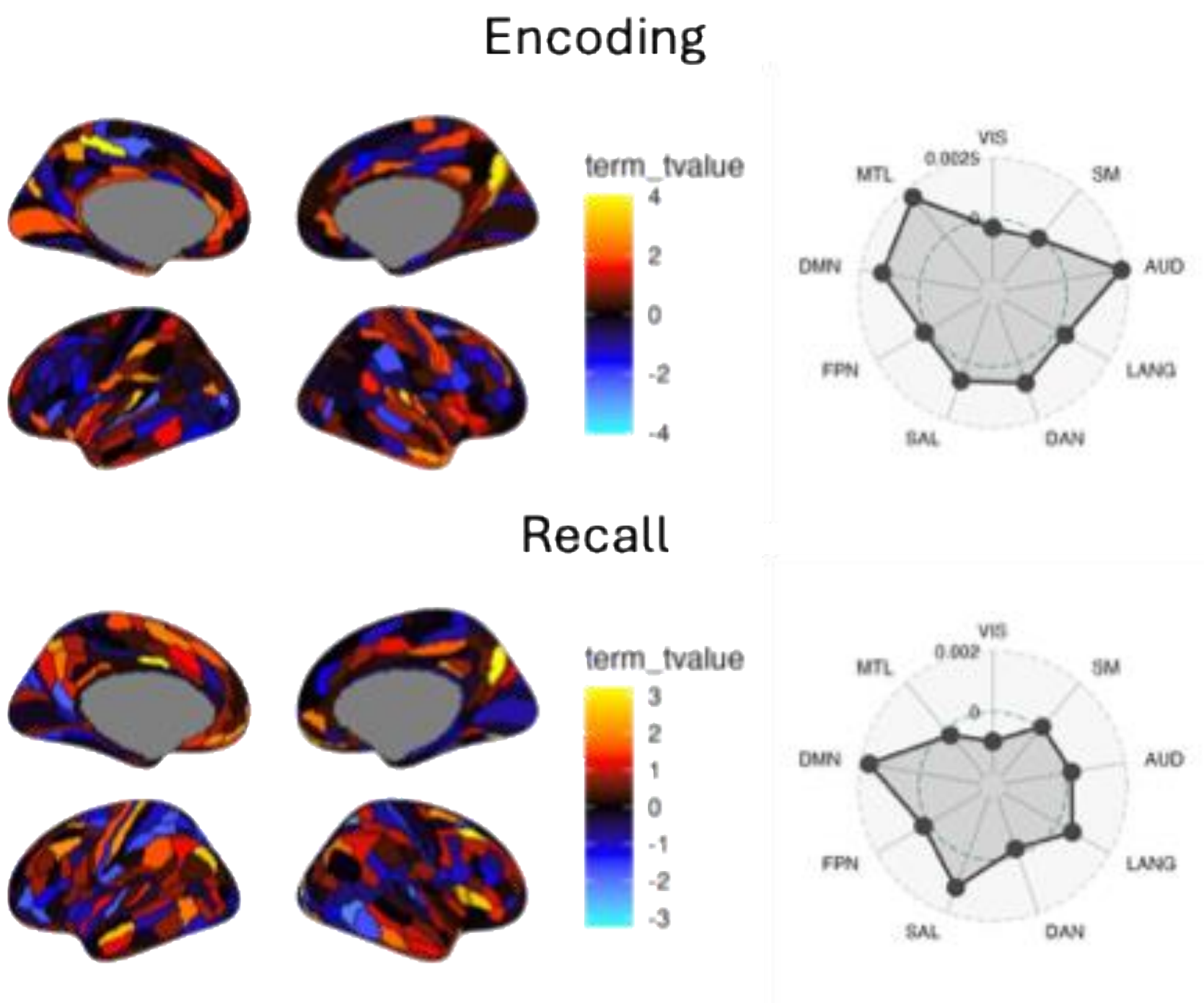

Figure 3. Unthresholded whole-brain ROI maps of t-values showing the relationship between ISPS and topic similarity using the HCP-MMP1.0 atlas (Glasser et al., 2016) on the left and radar plots of the mean slope for each network on the right.

2. The correct HyperTools reference (p. 6) is Heusser et al. (2018), not Pedregosa et al. (2011).

Thank you. We have fixed this citation.

Using methods developed by Heusser et al. (2021), we trained our model using these recall documents with scikit-learn 1.4.1(Pedregosa et al., 2011), called from HyperTools (Heusser et al., 2021)

3. In Figure 2A and B (p. 9) it would be nice to (a) use shared y-axes between the two panels and (b) either show individual data points (I realize there are many of them) or overlay the line plots on 2-D histograms (if showing individual data points is too messy). The two results in that figure are interesting, and clearly (from the stats reported on p. 9)

they are statistically reliable– but it's difficult to get a sense from the figures alone how the individual observations are distributed.

Thank you for this comment. Addressing it led to a series of changes in the analysis. First, upon plotting the histograms of the intrusions and details against topic similarity, we observed that the distribution of intrusions was zero inflated. This prompted further examination of model diagnostics revealing that a linear mixed model produced a poor fit for the data. To address this, we opted to divide the intrusions into two regressors, one which in a binary manner tracked intrusions (0 vs. more than 0) and a second that tracked intrusions in a graded manner when the intrusions were greater than 0.

To get a sense of the spread of the values I present the raw datapoints below, overlaid with the slopes estimated from linear mixed models. The intrusions plot only shows the graded effect (i.e. after removing the many zeros). Both intrusions and details recalled are the **sums** of these measures across the pair of participants that goes into the topic similarity.

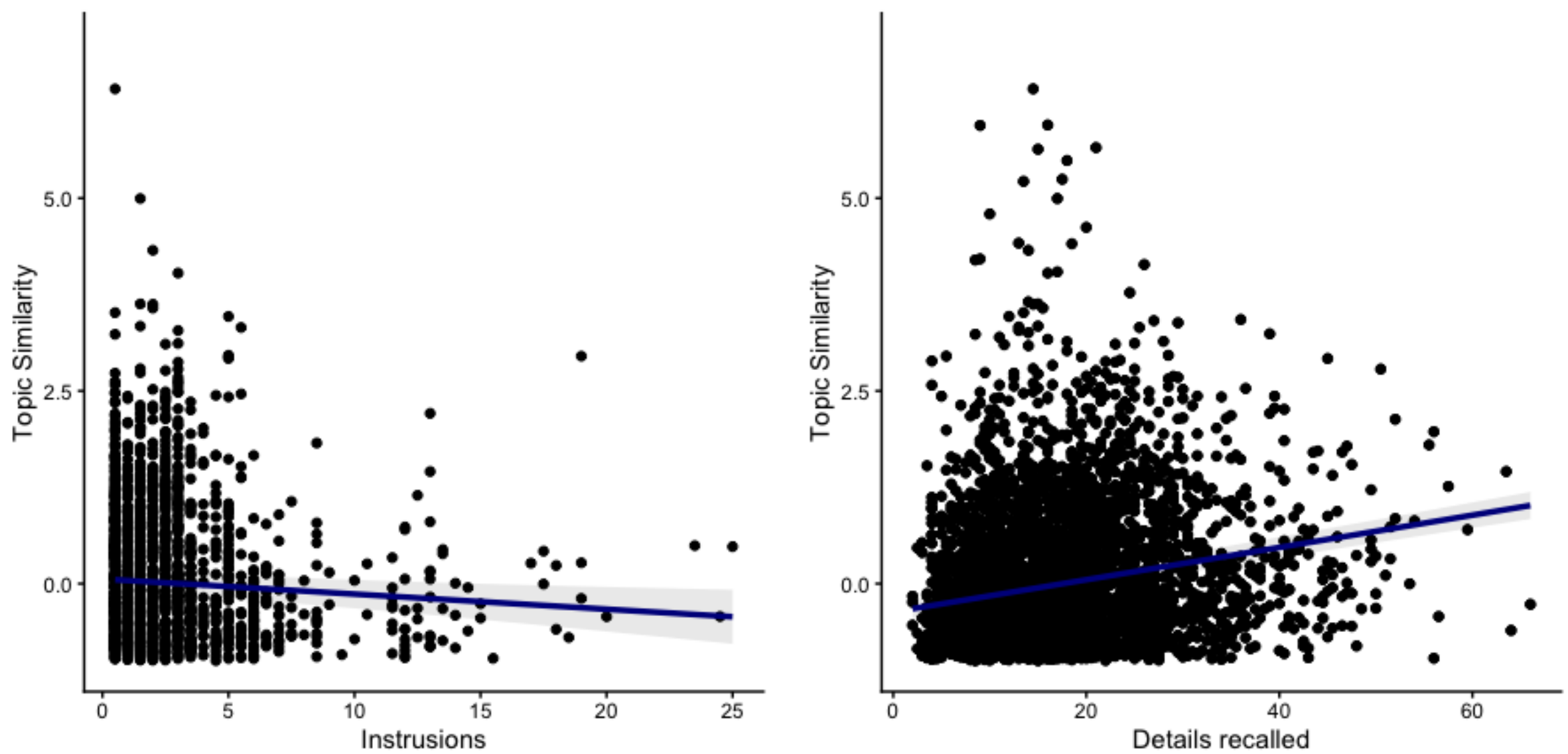

However, the model diagnostics we not ideal and, thus, to have better model fit, we used a Bayesian approach with a Student t distribution to estimating the relationship between topic similarity and details and intrusions. In the end we now report this relationship in the text, but no longer have a figure in the manuscript.

4. On p. 9 (bottom paragraph) the authors mention replicating the "topic similarity vs. encoding ISPS" analysis for a control ROI. However, I don't see stats for that control ROI (or associated figures) reported in the text. Similarly, in Fig. 3 (p. 10) it would be nice to also include the control ROI (in both panels) for comparison.

We have now included the statistics for the control ROI, the somatomotor cortex, in the text. It is displayed as the cyan point plot in Figure 3. All statistics are also provided in Supplemental Table 2.

5. One of the most fascinating “results” (sort of?) from the paper is the implication (p. 10) that ISPS at *encoding* better predicts recall topic similarity than ISPS at *recall*. But this is left implicit– the authors comment that “on the other hand, …recall activity…[is associated with topic similarity] only at uncorrected levels [in a subset of DMN regions]”. It would be helpful to present an explicit statistical test comparing the strength of ISPS-recall topic similarity associations for activity recorded during encoding vs. recall. A challenge (which the authors note on p. 12) is that participants have higher motion during recall, and therefore interpreting a weaker effect during recall requires deconfounding a true neural difference from motion artifacts. But perhaps it would be possible to regress out the effects of motion? In any case, even with caveats about interpretation, it would strengthen the story to include a direct comparison of the strengths of the associations between recall topic similarity and encoding vs. recall ISPS.

There was no significant interaction between topic similarity and phase (encoding vs. recall) in predicting ISPS for any of the ROIs. Beyond the simple

Reviewer #3 (Remarks to the Author):

I co-reviewed this manuscript with one of the reviewers who provided the listed reports. This is part of the Communications Psychology initiative to facilitate training in peer review and to provide appropriate recognition for Early Career Researchers who co-review manuscripts.

Reviewer #4 (Remarks to the Author):

Review of Kim
The current manuscript describes a novel analysis of behavioral and functional imaging data collected from a group of 24 individuals, towards the goal of exploring correlated event patterns in the default mode network (DMN) while watching a cartoon that overlap with the similarity in recalled events during that cartoon. The central premise of this work is the DMN represents event models, and that correlated patterns of activity across individuals during shared experiences are possible evidence of shared representation of high-level event features. The authors test this idea by combining NLP topic modeling based on recall transcriptions with a derived neural measure, “ISPS” (intersubject pattern similarity), which quantifies the correlation of mean activity for discrete events during two separate cartoons. The strength of the manuscript is driven by 2 main findings. First, greater similarity in recalled content was associated with stronger shared activation patterns at encoding. Second, topic similarity across participants increased as a function of the number of details recalled, supporting the hypothesis that similarity carries meaningful information. I find the manuscript to be well

designed and motivated, and have only one general comment, and a number of more minor comments towards a future revision.

Thank you for your kind comments on the manuscript.

One of the principal strengths and challenges for these data is suggestion that the DMN carries meaningful information from encoding forward to the recall of the narrative events. The data here successfully challenges many dominant views of DMNs as largely agnostic to information processing and more driven by factors like “state”, awareness, or attention. Nonetheless, the data presented here is nonetheless not conclusive on their argument, and alternative hypotheses are not rigorously entertained.

We have now noted the juxtaposition of our results in light of prior ideas relating DMN activity to internally directed thinking or attentional states and cited a recent paper discussing the expansion of the ideas.

These findings together point to representations in the DMN carrying event level information at both encoding and retrieval, suggesting a greater role in ongoing experience beyond just internally directed thought or attentional states (Zhou et al., 2025).

Relatedly, more clarity on what is concretely being represented by their analysis, beyond intrasubject similarity, would solidify the findings. Participants watched a 16 minute cartoon, and a topic modeling approach identified 5-55 topics that might show a common occurrence in free recall between participants. But we are not given information on what kind of cartoon was seen, what kinds of topics were identified, so it is difficult to link the data with the inferences made. While previously explained in Barnett et al. 2024, more details on the movie stimuli would be very useful, including a description of the characters, events, or contexts that comprise “event features”.

Thank you for your suggestions. We have now included more detail describing the videos and the identified topics.

Videos

These custom-made cartoons were made using Plotagon Studio (https://www.plotagon.com/desktop/). The movies depicted a continuous narrative, one following a stereotypical crime scene investigation style story and the other depicting a medieval fantasy style story. Each movie consisted of 14 events (mean 72s in length) which were defined by shifts in location, time, and character composition. These events were confirmed and identified by a sample of 62 online participants from Barnett et al (2024) who were instructed to watch one of the videos and press the spacebar when they felt that "one meaningful event had ended and another had begun" in line with prior work in the field (Ben-Yakov & Henson, 2018; Sargent et al., 2013). The resulting timecourse of button presses from each participant was combined to form a distribution which was modelled with a kernel density estimate. Peaks in the density confirmed the intended event boundaries designed in the stimuli (and identified an additional

boundary in each stimulus). These were used as "event boundaries" in Barnett et al (2024). Here, an "event" is defined as the time between one event boundary and the next.

Topics

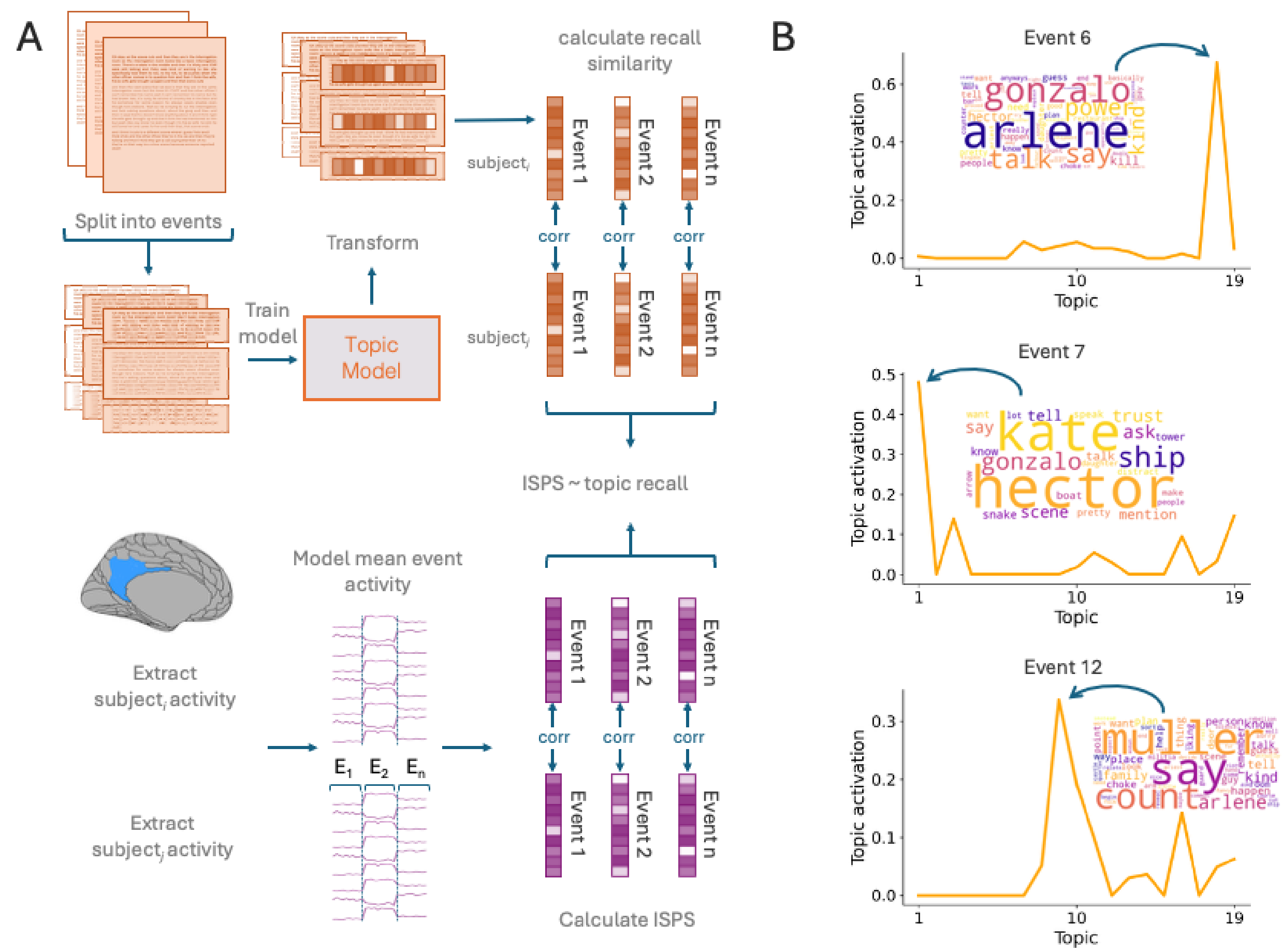

***Figure 1. A**. Overview of analysis pipeline. TOP: The preprocessed recall transcripts for each participant were divided into separate documents for each event. The collection of documents was then used to train a topic model to identify latent topics. Each event from each participant was then transformed into a vector of topic proportions that reveal the combination of topics present in the recalled event using the trained topic model. To calculate topic similarity, we correlated the vector of topic proportions from matching events between participants and Fisher z-transformed the correlation. BOTTOM: To examine intersubject pattern similarity (ISPS), multivoxel activity was extracted from each ROI and was modeled to find the mean multivoxel pattern for each event and each participant. We then correlated these multivoxel patterns for matching events between pairs of participants and Fisher-z transformed the correlation to get ISPS. **B.** Plots showing the average topic embeddings for three example events along with word clouds highlighting the top words that contribute to those latent topics (larger words contribute more to topic activation). Of note, topics often consisted of present or mentioned characters*

along with spatial location or topics of conversation (e.g. Hector, Kate, Ship, for event 7 that depicts those characters on a boat).

More details on the participant pool are necessary (age, # of M/F, compensation, IRB approval).

We have now added these additional details.

Dataset

Our dataset is a re-analysis of Barnett et al (2024) which consisted of 24 healthy, right-handed young adults (mean age = 23.5 years; $N_{Females}$ = 14) with no history of neurological issues who watched two 16.5 minute custom-made cartoon movies and then freely recalled them in the scanner (Barnett et al., 2024). These custom-made cartoons were made using Plotagon Studio (https://www.plotagon.com/desktop/). The movies depicted a continuous narrative, one following a stereotypical crime scene investigation style story and the other depicting a medieval fantasy style story. Each movie consisted of 14 events (mean 72s in length) which were defined by shifts in location, time, and character composition. These events were confirmed and identified by a sample of 62 online participants from Barnett et al (2024) who were instructed to watch one of the videos and press the spacebar when they felt that "one meaningful event had ended and another had begun" in line with prior work in the field (Ben-Yakov & Henson, 2018; Sargent et al., 2013). The resulting timecourse of button presses from each participant was combined to form a distribution which was modelled with a kernel density estimate. Peaks in the density confirmed the intended event boundaries designed in the stimuli (and identified an additional boundary in each stimulus). These were used as "event boundaries" in Barnett et al (2024). Here, an "event" is defined as the time between one event boundary and the next. Participants were compensated $20 an hour for their time and the study was approved by the institutional review board of the University of California at Davis (IRB #1352490 & IRB # 637028) and the University of Toronto (45451).

Our dataset is a re-analysis of Barnett et al (2024) which consisted of 24 healthy, right-handed young adults (mean age = 23.5 years; $N_{Females}$ = 14) with no history of neurological issues who watched two 16.5 minute custom-made cartoon movies and then freely recalled them in the scanner (Barnett et al., 2024).

Participants were compensated $20 an hour for their time and the study was approved by the institutional review board of the University of California at Davis (IRB #1352490 & IRB # 637028) and the University of Toronto (45451).

MRI procedure/acquisition details on the recall period are unclear. What were the run lengths for the recall runs? Did scanner noise interfere with transcription? There are no details about the MRI acquisition for this part of the paradigm, even though the fMRI data during the recall period comprises the main focus of the analysis.

We have included additional information on the procedure during recall.

Run lengths varied between participants as they each had varying lengths of recall. We now include this range in the methods.

Participants were allowed to recall until they could no longer recall any additional details. Thus, run lengths varied between participants from (270s to 1016s; mean = 688s)

Scanner noise was mitigated by the bidirectional noise-cancelling features of the FOMRI-III.

" Participants verbally recalled into an MRI compatible microphone (Optoacoustics FOMRI-III; https://www.optoacoustics.com/medical/fomri-iii) with bidirectional noise-cancelling to mitigate scanner noise."

MRI acquisition parameters have been added

"… functional images were acquired using a gradient EPI sequence (TR = 1220 ms; TE = 24 ms; field of view = 192 mm2; image matrix = 64 × 64; flip angle = 67°; multiband factor = 2; 38 interleaved axial slices, voxel size = 3 mm3 isotropic)."

Ln 143: The authors estimate the mean activity across an event, and then calculated the similarity in the activity across events; how many events are used to generate the Pearson's correlation? How long are these events?

Each movie contained 14 events that varied in length from 22s to 137s (avg. 72s), resulting in 28 event patterns per person. Matching event patterns were correlated between pairs of participants. With 24 participants there are 276 unique pairs, and with 28 events that results in 7728 event correlations. However, not all of these end up being used because if the event is not subsequently recalled by a participant then we cannot calculate their recall similarity to other participants. This is described in the response below.

More broadly, the manuscript needs to make clear that the ISPS analysis was conducted during recall, not encoding.

ISPS analysis on fMRI data was calculated during both encoding and recall. However, event patterns during encoding only will be used in the statistical models for events that are subsequently recalled (because if the event is not recalled, then we cannot estimate the similarity of the recalled transcripts). We have clarified this in the methods.

Next, a separate set of linear mixed models were fit to predict ISPS from topic similarity for each ROI at both encoding and retrieval with a fully crossed random effect approach…"

"...While participants viewed all events during encoding, in the linear mixed models to predict encoding ISPS only events that are subsequently recalled by both pairs of participants are

included in the model because if an event is not recalled, it is not possible to calculate topic similarity.

This is very subjective, but Figure 1 (bottom) implies that event boundaries are a strong component of the data, but these are never explicitly modeled in the current data. Furthermore, it could be made clearer that the authors are averaging across discrete periods of the timeseries, and that TR-to-TR fluctuations (i.e., typical FC) are not at play here. The title specifies “Model mean event activity”, but this is ambiguous. Authors may wish to adjust the timeseries depiction to de-emphasize this.

Thank you for this suggestion. We have de-emphasized the event boundaries and emphasized the event to better depict that that what we are doing is calculating the mean multivoxel event pattern.

Signed,
Simon Davis
Indiana University

Reviewer #1 (Remarks to the Author):

The authors have addressed some of the previous concerns and added a few methodological details. However, several issues remain that should be clarified or corrected.

1. In the last paragraph of the Introduction (p. 4), the authors revised the text to indicate that the current study reanalyzes a previously published dataset. However, the revision seems to unnecessarily remove most of the information describing that prior study. Even if the dataset has been published previously, the introduction should still provide at least a brief description of the original study (e.g., its design).

The journal had asked for methodology to be removed from the final paragraph of the introduction but we agree that we were overzealous in removing parts of the methodology that pertain to an already published paper and have re-entered it.

In this study, we used natural language processing and fMRI to examine how multivariate event patterns in the DMN are shared during encoding and recall, depending on the degree of similarity in which participants remembered events. To do so, we re-analyzed data from Barnett et al (2024) in which 24 participants watched and verbally recalled two cartoon movies during fMRI scanning. We hypothesized that people who recall events more similarly to each other would show stronger intersubject pattern similarity in DMN regions, particularly the mPFC and PMC – regions that have been heavily implicated in representing higher order abstractions of events (Audrain & McAndrews, 2022; Baldassano et al., 2018; Reagh & Ranganath, 2018; Zadbood et al., 2022). We also examined how recall similarity related to simpler scoring measures of memory content such as the total number of details recalled and the number of intrusions, expecting greater similarity in recall when participants recalled many true details and few intrusions.

2. In the section “Effects beyond the DMN” (pp. 14-15), the reported statistical tests are inconsistent. Some regions are reported with one-sided tests (e.g., DMN, MTL), while some are reported with two-sided tests (e.g., AUD). It is unclear why different testing directions are used across regions. Unless there is a clear a priori justification for using one-sided tests for specific networks, the statistical test should be applied consistently.

We had a priori region to suspect the DMN would have a greater contribution compared to other networks in the brain. However, the reviewer is correct to point out that this was not conveyed well. We have made this more clear in the revised manuscript.

Methods

To determine whether the DMN had specificity in this relationship, we used a permutation test on the whole brain data. We calculated the difference in the mean slope estimates between the DMN and all other networks. We then produced a null distribution by

randomly re-labelling each ROI and recalculating the differences 10,000 times. Significance was assessed by observing the proportion of times the true difference was greater or smaller than differences in the null distribution. Based on prior work (J. Chen et al., 2017; Reagh & Ranganath, 2023), we anticipated a positive difference in favor of the DMN and, thus, performed a one-sided test.

3. On page 8, the manuscript states that an optimal number of topics (K) was selected, but the text still includes the sentence, “The resulting topic similarity values were averaged across the range of topic numbers”, which is confusing. Does this mean that the authors still average across different values of K even after selecting the optimal K?

Thank you for pointing this out. This sentence should have been deleted as there is no longer a range of topic numbers.

In addition, the plots showing model performance as a function of the number of topics should be reported as a supplementary figure. The manuscript also does not report the variance associated with different random seeds (this could be shown as error bars in the supplementary figure).

Thank you for pointing this out. We had missed this in the original revisions. Upon inspecting the variance across the random seeds, we realized that using a Pearson’s correlation to calculate the similarity between topic vectors was problematic. This was because some iterations resulted in topic vectors for events that were heavily skewed by a single topic and violated required assumptions. Spearman correlations offered a nonparametric approach that is robust to scale and distributional differences. This change led to largely similar results but some differences which we will outline. To provide clarity on the variance associated with random seeds we calculated the intraclass correlation between runs and found high consistency, ICC = 0.97. In addition to this change, reviewer 2 requested examining whether the relationship between topic similarity and ISPS differed between encoding and recall. To examine this, we predicted topic similarity from both encoding ISPS and recall ISPS in a single linear mixed model and then used a Wald Chi Square test to test the difference between the slopes. To keep this consistency across our models we used ISPS to predict topic modeling throughout which did not change the significance of results.

Methods

To ensure that our estimates of topic similarity are not sporadic, we also fit the model 100 times using a new random seed for the optimal K number of topics and averaged the similarity to bootstrap our findings and used the average topic similarity. Intraclass correlation was calculated across the 100 iterations to determine the impact of the random seeds.

Results

Optimal number of topics

Pattern classifiers were able to accurately classify which event was being described in left out data for every value of K (chance = 1/14, accuracy range = 24% – 56%). We observed that classifier accuracy increased with higher numbers of topics. The increase in accuracy showed diminishing returns between 19 and 31 topics for the two narratives. We then inspected the content of the topics identified by the LDA. We observed that beyond K = 19, the models produced degenerate topics characterized by semantically unrelated, low-frequency words (often appearing alphabetically ordered) with near uniform word distributions consistent with topics that were weakly constrained by the data. As such, K = 19 was selected as the optimal number of topics as it could significantly predict the event being described on left out data and was free from degenerate topics. Across the 100 iterations with differing random seeds, intraclass correlation was very high, ICC = 0.97.

When re-running the analyses with the Spearman correlation topic similarity we observed much more widespread associations between topic similarity and intersubject pattern similarity at encoding, but there was still specificity to the DMN at recall. This was updated in the results, figure, and discussion sections

4. Remaining minor issues in the manuscript:

-Page 7, line 207: The sentence “we divided the recall transcript into separate based on…” is missing the word “segments.” This issue was pointed out previously but remains unaddressed.

Thank you for pointing this out to us again. It is now corrected.

-Pages 9-10, the model description still contains an error that was pointed out before but not addressed. The manuscript states: “we fit a set of models that included encoding intersubject pattern similarity and recalled details … to predict intersubject pattern similarity.” The predictor should be 'topic similarity'.

Thank you for pointing this out. We have re-worked this section based on responses to reviewer 2.

To determine the unique effect of intersubject pattern similarity on topic similarity, we fit a separate set of models that included encoding intersubject pattern similarity and recalled details (for both participants) in the model to predict topic similarity.

-There is an inconsistency in the reported degrees of freedom. On pages 13–14, the revised manuscript reports t(3994), but Supplemental Table 2 and Table 3 report df = 3993.

3994 was correct, the supplemental tables were not accordingly updated. We have now fixed this

-Page 5, line 144: The sentence "Thus, run lengths varied between participants from (108s to 1016s; mean = 688s)" is ungrammatical and should be revised.

Thank you. This has now been corrected.

Thus, run lengths varied between participants from 108s to 1016s (mean = 688s).

-The response to Reviewer 2, Comment 5 appears to be truncated in the response letter. The text ends with "Beyond the simple".

Thank you for pointing out this mistake. We have addressed it below.

Reviewer #2 (Remarks to the Author):

The authors have done a nice job with their revision. I have just a few minor suggestions:

1. The HyperTools ref is here (note: 2018, not 2021): https://www.jmlr.org/papers/v18/17-434.html

Thank you for the correct citation. This has now been updated.

2. I suggest including the scatterplots showing topic similarity vs. intrusions and details recalled in supplemental materials. And given the shapes of those distributions, I'd suggest toning down the associated claims (using your Bayesian/t-distribution approach) accordingly. They look a bit messy, so that should be acknowledged in some form.

Upon inspecting the variance across the random seeds, we realized that using a Pearson's correlation to calculate the similarity between topic vectors was problematic. This was because some iterations resulted in topic vectors for events that were heavily skewed by a single topic and violated required assumptions. Spearman correlations offered a noparametric approach that is robust to scale and distributional differences. This change led to largely similar results but some differences which we will outline. Because of this change, the model diagnostics using linear mixed models improved significantly and for consistency, we opted to return to the linear mixed model approach and away from the Bayesian approach. The overall effects remained largely similar as can be seen below in the text and figures. The figures present a more coherent distribution of topic similarity values.

Similarity of topic patterns and scored details

We tested if recall similarity measured by topic similarity captured similar content as human raters. Linear mixed modeling revealed that event topic similarity was positively associated with

the number of event details that were recalled (Supplemental Figure 1), ($t(3996) = 15.4$, $p < .0001$, $\beta = .06$, S.E. = .002). Next, we observed that the binary presence of intrusions was significantly associated with topic similarity in a positive fashion which was surprising ($t(3996) = 2.36$, $p = .02$, mean $\beta = .01$, S.E. = .004). Conversely, when looking at the number of intrusions as a continuous variable (excluding trials with 0 intrusions as this would create a zero inflated distribution), there was a significant negative relationship between the number of intrusions topic similarity ($t(1960) = -3.03$, $p = .003$, $\beta = -.0.09$, S.E. = .003. Given these contradictory findings, we examined whether these effects endured after accounting for the number of true event details (perhaps participants who provided many verifiable event details were also more likely to contribute intrusions). When re-running the linear mixed models to predict topic similarity from both the number of event details and the binary predictor of intrusions, the number of details remained significant ($t(3995) = 25.7$, $p <.0001$, $\beta = .06$, S.E. = .002), but the binary intrusions predictor was not ($t(3995) = -1.05$, $p = .3$, $\beta = -.004$, S.E. = .003). When re-running the linear mixed models to predict topic similarity from both the number of event details and the number of intrusions, both the number of details ($t(1959) =16.7$, $p <.0001$, $\beta = .05$, S.E. = .002) and the number of intrusion ($t(1959) = -6.03$, $p <.0001$, $\beta = -.02$, S.E. = .002) remained significant. This demonstrates that the topics produced from the topic models are sensitive to the content of retrieval in an event-specific manner. When participants recall more true details and fewer intrusions, they show greater similarity.

We will include these figures in the supplemental information file.

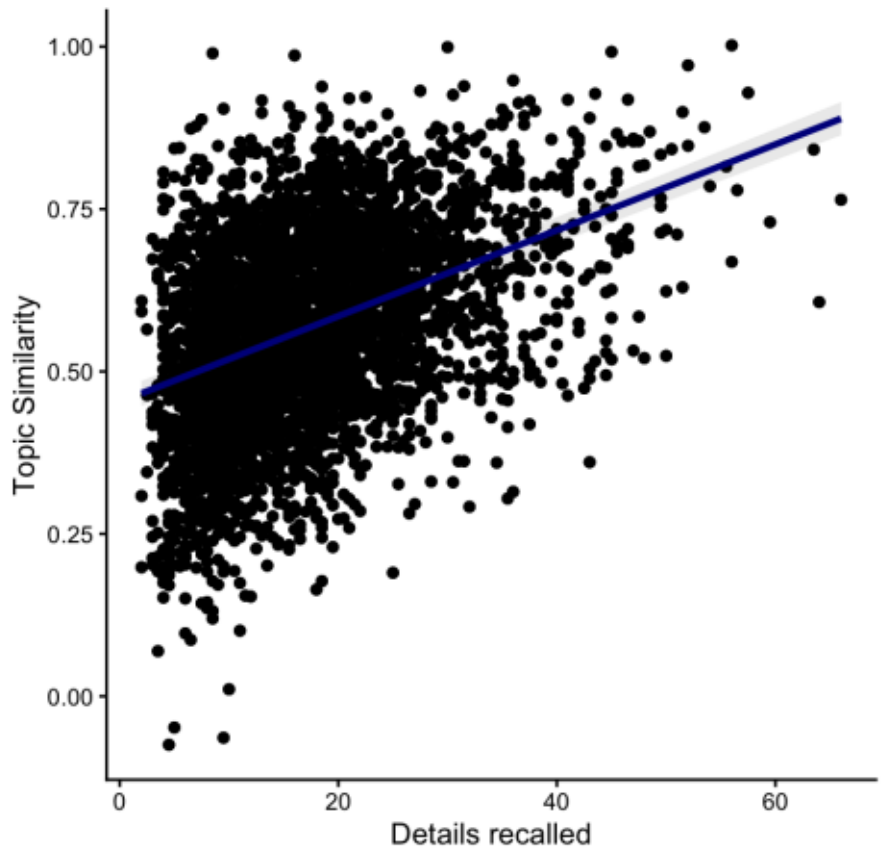

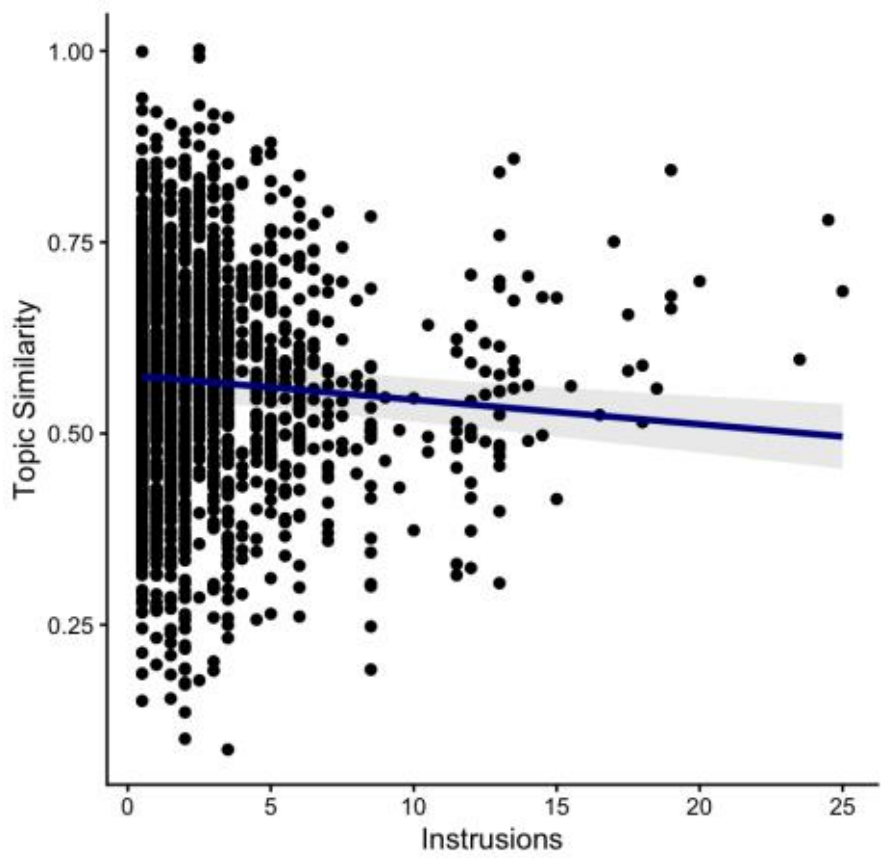

Supplemental Figure 1. Top. Scatterplot and regression line showing the relationship between the total details recalled between pairs and the topic similarity between corresponding pairs. Bottom. Scatterplot and regression line showing the relationship between the total intrusions made between pairs and the topic similarity between corresponding pairs.

3. Your response to my 5th "concern" (listed under R2) appears to have been cut off (ends at "Beyond the simple" with no rest of the sentence). But, extrapolating from what I can see: if there is no interaction between topic similarity and phase, that should be made clear in the manuscript and to report those stats.

Apologies for the cut-off sentence. To better completely answer this point, we want to re-mention that topic similarity was recalculated using Spearman instead of Pearson correlations. Next, to best characterize the effect of encoding ISPS vs. recall ISPS on topic similarity we have re-organized the linear mixed models to make topic similarity the dependent variable and encoding ISPS and recall ISPS the predictors. We then used a Wald test to examine whether encoding ISPS was different in its slope compared to recall ISPS. This resulted in a marginal difference in PMC predictability of topic similarity between encoding and retrieval. We have reported this now.

METHODS

Next, a separate set of linear mixed models were fit to predict topic similarity from multivoxel intersubject pattern similarity for each ROI at both encoding and retrieval with a fully crossed random effect approach(G. Chen et al., 2017) using lmer (Bates et al., 2015) in R 4.4.1 (R Core Team, 2021). In this structure, each intersubject pattern similarity value in the model comes from a pair of participants i and j, where $i \neq j$. Random effects are modeled for both the first and second participants in the pairing. Since the random effects are fully crossed, each participant appears in both the first random effect and the second random effect. However, this also leads to a data doubling in the model which is later corrected by discounting the degrees of freedom in the standard error of a term from the model and the degrees of freedom for the t-test and p-value calculations of those terms in line with G. Chen et al. 2017. This is done by dividing

the number of observations by 2 and subtracting the number of fixed effects and using this value to replace the inflated degrees of freedom. The t-test statistics are recalculated using the fixed effects from the model and the updated standard error. Since topic similarity is calculated by a pair of participants, participant i and participant j, a linear mixed model was fit to predict topic similarity from the details recalled from participant i and the details recalled from participant j in line with G. Chen et al. 2017. Each participant contributes to both regressors for the details recalled for participant i and the details recalled from participant j, resulting in an identically estimated slope. To determine whether the unique effect of intersubject pattern similarity on topic similarity, we fit a separate set of models that included encoding intersubject pattern similarity and recalled details (for both participants) in the model to predict topic similarity. While participants viewed all events during encoding, in the linear mixed models to predict encoding intersubject pattern similarity only modelled events that are subsequently recalled by both pairs of participants because if an event is not recalled, it is not possible to calculate topic similarity.

$$topicModelingSimilarity \sim encISPS + detailsRecall_{s_i} + detailsRecall_{s_j} + (1|S_i) + (1|S_j)$$

$$topicModelingSimilarity \sim recISPS + length_{s_i} + length_{s_j} + (1|S_i) + (1|S_j)$$

Also, when predicting topic similarity from recall intersubject pattern similarity, we included the number of TRs that elapsed as a participant described an event (i.e. the length of time they took recalling an event) as a regressor of no interest to ensure that relationships between topic similarity and intersubject pattern similarity are not simply due to having more data to estimate multivoxel patterns. Significance was assessed at p < .05, FDR correcting for the number of predefined ROIs and all predictors were mean centered. The exploratory whole-brain analysis is presented unthresholded.

Next, we sought to examine whether there was a difference in the association between encoding intersubject pattern similarity versus recall intersubject pattern similarity with topic similarity. We included both pattern similarity measures in the same model to predict topic similarity and used a Wald Chi Square test to test the difference between the slopes. Significance was assessed at p < .05, FDR correcting for the number of predefined ROIs

RESULTS

To examine whether intersubject pattern similarity varied in its relationship to topic similarity based on whether it was calculated at encoding or retrieval, we fit a linear mixed model to predict topic similarity from both encoding intersubject pattern similarity and recall intersubject pattern similarity, controlling for the number of recalled details. A Wald Chi squared test was used to determine whether the predictors significantly varied. After correcting for multiple comparisons, we did not see any significant differences between the predictors, but there

was a trend showing that PMC intersubject pattern similarity was a marginally stronger predictor of topic similarity at encoding compared to recall ($\chi^2 = 7.05$, $p = .079$).

Reviewer #3 (Remarks to the Author):

I co-reviewed this manuscript with one of the reviewers who provided the listed reports. This is part of the Communications Psychology initiative to facilitate training in peer review and to provide appropriate recognition for Early Career Researchers who co-review manuscripts.

Reviewer #4 (Remarks to the Author):

The revised manuscript represents a thorough revision of the analysis and presentation of the authors application of NLP tools to an fMRI encoding and recall dataset. The authors have addressed all extant concerns, including more details on the MRI acquisition and task paradigm, greater detail on what comprises an "event" in their analysis, a small clarification on the role of the DMN, and more minor modifications requested throughout. The more refined version of this manuscript is a pleasure to read.

Thank you very much for the kind words.
