# Supplementary material for: Natural language processing captures memory content associated with shared neural patterns at encoding and retrieval
Source: Commun Psychol. 2026 Jun 4;4:124. doi: 10.1038/s44271-026-00481-0 (PMC13547101; doi:10.1038/s44271-026-00481-0)
Supplement: Supplementary file 2 [file 44271_2026_481_MOESM2_ESM.pdf]

Supplemental Table 1. Topic similarity and Encoding ISPS

Glasser ROI name	hemisphere	ROI Combo
RSC	L	PMC
7m	L	PMC
POS1	L	PMC
v23ab	L	PMC
d23ab	L	PMC
31pv	L	PMC
31pd	L	PMC
31a	L	PMC
RSC	R	PMC
7m	R	PMC
23d	R	PMC
v23ab	R	PMC
d23ab	R	PMC
31pv	R	PMC
31pd	R	PMC
31a	R	PMC
d32	L	dmPFC
9m	L	dmPFC
8BL	L	dmPFC
9p	L	dmPFC
10d	L	dmPFC
9a	L	dmPFC
d32	R	dmPFC
9m	R	dmPFC
8BL	R	dmPFC
9p	R	dmPFC
10d	R	dmPFC
9a	R	dmPFC
TGd	L	Lat temp
TE1a	L	Lat temp
TE2a	L	Lat temp
STSva	L	Lat temp
TE1m	L	Lat temp
TGd	R	Lat temp
TE1a	R	Lat temp
TE2a	R	Lat temp

STSva	R	Lat temp
TE1m	R	Lat temp
a24	L	vmPFC
p32	L	vmPFC
10r	L	vmPFC
47m	L	vmPFC
10v	L	vmPFC
10pp	L	vmPFC
OFC	L	vmPFC
25	L	vmPFC
s32	L	vmPFC
pOFC	L	vmPFC
a24	R	vmPFC
p32	R	vmPFC
10r	R	vmPFC
47m	R	vmPFC
10v	R	vmPFC
10pp	R	vmPFC
OFC	R	vmPFC
25	R	vmPFC
s32	R	vmPFC
pOFC	R	vmPFC
PreS	L	MTL
ProS	L	MTL
PeEc	L	MTL
PHA1	L	MTL
PHA3	L	MTL
PHA2	L	MTL
PreS	R	MTL
ProS	R	MTL
PeEc	R	MTL
PHA1	R	MTL
PHA3	R	MTL
TF	R	MTL
PHA2	R	MTL
PCV	L	POS_PreC
7Pm	L	POS_PreC
7Am	L	POS_PreC
7PL	L	POS_PreC

DVT	L	POS_PreC
POS2	L	POS_PreC
PCV	R	POS_PreC
7Pm	R	POS_PreC
POS1	R	POS_PreC
7Am	R	POS_PreC
7PL	R	POS_PreC
TPOJ3	L	vLP
PGp	L	vLP
TPOJ3	R	vLP
PGp	R	vLP
PGs	L	dLP
PGs	R	dLP
PGi	R	dLP
PFM	L	aLP
PGi	L	aLP
4	L	SMC
3b	L	SMC
5m	L	SMC
5L	L	SMC
24dd	L	SMC
24dv	L	SMC
1	L	SMC
2	L	SMC
3a	L	SMC
6d	L	SMC
6mp	L	SMC
6v	L	SMC
OP4	L	SMC
OP1	L	SMC
OP2-3	L	SMC
FOP2	L	SMC
Ig	L	SMC
4	R	SMC
3b	R	SMC
5m	R	SMC
5L	R	SMC
24dd	R	SMC
24dv	R	SMC

1	R	SMC
2	R	SMC
3a	R	SMC
6d	R	SMC
6mp	R	SMC
6v	R	SMC
OP4	R	SMC
OP1	R	SMC
OP2-3	R	SMC
FOP2	R	SMC
Ig	R	SMC

ATL, anterior temporal lobe; aLP, anterior lateral parietal cortex; dLP, dorsal lateral parietal cortex; dmPFC, dorsomedial prefrontal cortex; MTL, medial temporal lobe; PMC, posterior medial cortex; PreC, precuneus; SMC, somatomotor; vLP, ventral lateral parietal; vmPFC, ventromedial prefrontal cortex.

Supplemental Table 2. Topic similarity and Encoding ISPS

Relationship between Topic similarity and Encoding ISPS (accounting for details) DF = 3994

ROI	slope (s.e.)	t-value	p-value	FDR corrected
PMC	.011 (.002)	4.92	.0000009	**.000009**
dmPFC	.009 (.0002)	3.84	.0001	**.0006**
ATL	.005 (.0002)	2.42	.02	**.03**
vmPFC	.005 (.002)	2.26	.02	**.04**
MTL	.005 (.002)	2.21	.03	**.04**
PreC	.005 (.002)	2.4	.00	**.03**
vLP	.005 (.002)	2.03	.04	.05
dLP	.002 (.002)	.64	.52	.52
aLP	.003 (.002)	1.13	.26	.28
SMC	.005 (.002)	2.56	.01	**.03**

ATL, anterior temporal lobe; aLP, anterior lateral parietal cortex; dLP, dorsal lateral parietal cortex; dmPFC, dorsomedial prefrontal cortex; MTL, medial temporal lobe; PMC, posterior

medial cortex; PreC, precuneus; SMC, somatomotor; vLP, ventral lateral parietal; vmPFC, ventromedial prefrontal cortex.

Supplemental Table 3. Topic similarity and Recall ISPS

Relationship between Topic similarity and Recall ISPS (accounting for details) DF = 3994

ROI	slope (s.e.)	t-value	p-value	FDR corrected
PMC	.007 (.002)	3.23	.0012	**.012**
dmPFC	.004 (.002)	1.74	.08	.16
ATL	.005 (.002)	2.58	.01	**.033**
vmPFC	.006 (.002)	2.88	.004	**.02**
MTL	.002 (.002)	.92	.36	.44
PreC	.002 (.002)	.73	.47	.47
vLP	.002 (.002)	.94	.35	.44
dLP	.003 (.002)	1.36	.17	.29
aLP	.002 (.002)	.86	.39	0.44
SMC	.004 (.002)	1.87	.06	0.15

ATL, anterior temporal lobe; aLP, anterior lateral parietal cortex; dLP, dorsal lateral parietal cortex; dmPFC, dorsomedial prefrontal cortex; MTL, medial temporal lobe; PMC, posterior medial cortex; PreC, precuneus; SMC, somatomotor; vLP, ventral lateral parietal; vmPFC, ventromedial prefrontal cortex.

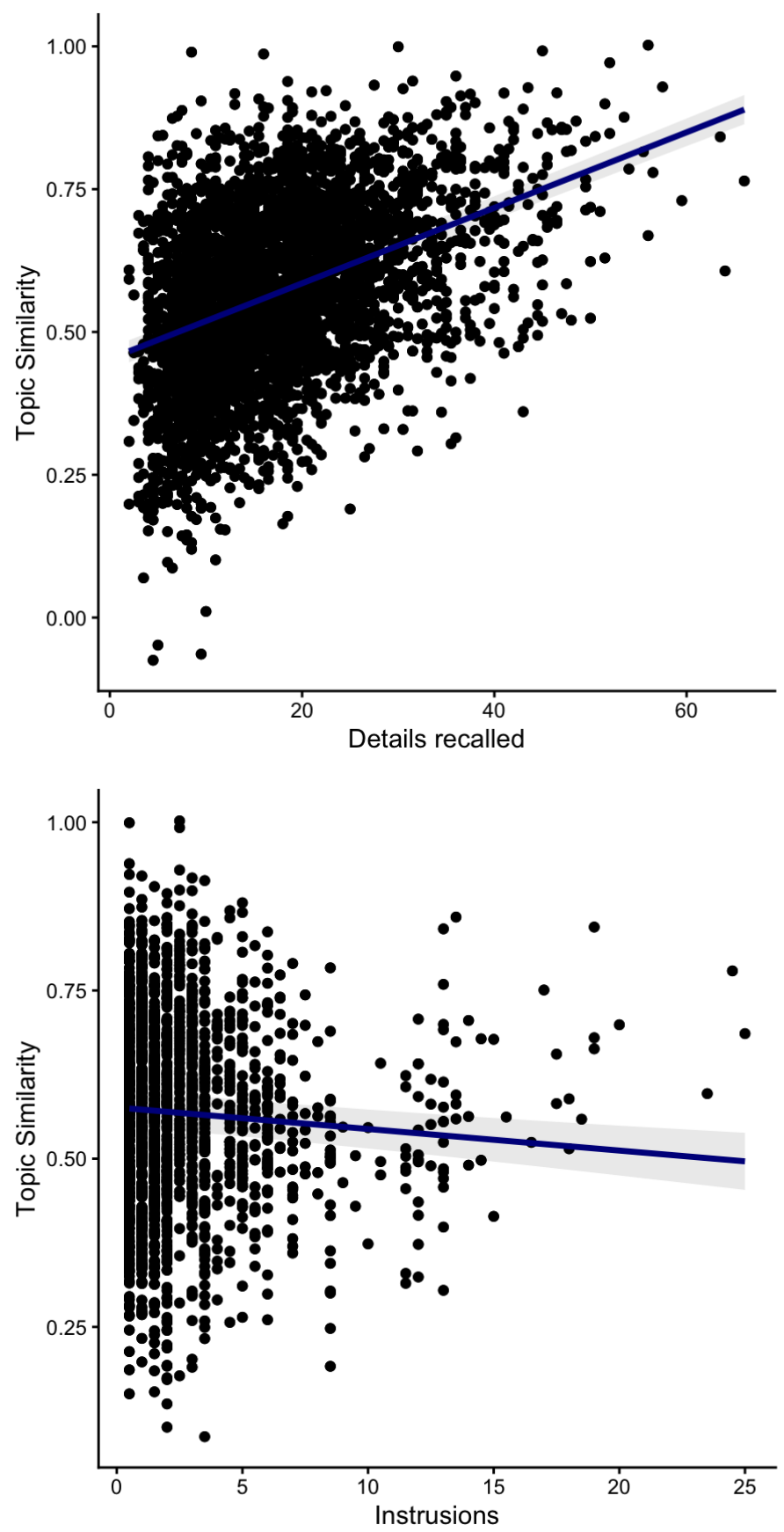

Supplemental Figure 1. Top. Scatterplot and regression line showing the relationship between the total details recalled between pairs and the topic similarity between corresponding pairs. Bottom. Scatterplot and regression line showing the relationship between the total intrusions made between pairs and the topic similarity between corresponding pairs.
